# Insights into methionine *S*-methylation in diverse organisms

**DOI:** 10.1038/s41467-022-30491-5

**Published:** 2022-05-26

**Authors:** Ming Peng, Chun-Yang Li, Xiu-Lan Chen, Beth T. Williams, Kang Li, Ya-Nan Gao, Peng Wang, Ning Wang, Chao Gao, Shan Zhang, Marie C. Schoelmerich, Jillian F. Banfield, J. Benjamin Miller, Nick E. Le Brun, Jonathan D. Todd, Yu-Zhong Zhang

**Affiliations:** 1 Frontiers Science Center for Deep Ocean Multispheres and Earth System & College of Marine Life Sciences, Ocean University of China, Qingdao, China; 2 State Key Laboratory of Microbial Technology, Marine Biotechnology Research Center, Shandong University, Qingdao, China; 3 Laboratory for Marine Biology and Biotechnology, Pilot National Laboratory for Marine Science and Technology, Qingdao, China; 4 School of Biological Sciences, University of East Anglia, Norwich Research Park, Norwich, UK; 5 Department of Earth and Planetary Science, Innovative Genomics Institute Building, University of California, Berkeley, Berkeley, CA USA; 6 Centre for Molecular and Structural Biochemistry, School of Chemistry, University of East Anglia, Norwich Research Park, Norwich, UK

**Keywords:** Environmental microbiology, X-ray crystallography, Enzyme mechanisms, Transferases

## Abstract

Dimethylsulfoniopropionate (DMSP) is an important marine anti-stress compound, with key roles in global nutrient cycling, chemotaxis and, potentially, climate regulation. Recently, diverse marine *Actinobacteria*, *α-* and *γ-proteobacteria* were shown to initiate DMSP synthesis via the methionine (Met) *S*-methyltransferase enzyme (MmtN), generating *S*-methyl-Met (SMM). Here we characterize a roseobacterial MmtN, providing structural and mechanistic insights into this DMSP synthesis enzyme. We propose that MmtN uses the proximity and desolvation mechanism for Met *S*-methylation with two adjacent MmtN monomers comprising the Met binding site. We also identify diverse functional MmtN enzymes in potentially symbiotic archaeal *Candidatus* Woesearchaeota and Candidate Phyla Radiation (CPR) bacteria, and the animalcule *Adineta steineri*, not anticipated to produce SMM and/or DMSP. These diverse MmtN enzymes, alongside the larger plant MMT enzyme with an N-terminus homologous to MmtN, likely utilize the same proximity and desolvation mechanism. This study provides important insights into the catalytic mechanism of SMM and/or DMSP production, and proposes roles for these compounds in secondary metabolite production, and SMM cycling in diverse organisms and environments.

## Abstract (web summary)

S-methyl methionine (SMM) is a key molecule in production of dimethylsulfoniopropionate (DMSP), an important marine anti-stress compound, with roles in global nutrient cycling. Here, the authors determine the mechanism of SMM synthesis and uncover unexpected roles for SMM in archaea, CPR bacteria and animals.

## Introduction

The sulfur-containing zwitterion dimethylsulfoniopropionate (DMSP) is produced at petagram levels in Earth’s oceans annually^1^, constituting up to ~10% of the fixed carbon^2^. DMSP production was thought to be a largely photic and oxic process, carried out primarily by algae, corals and a few plants^3^. However, heterotrophic bacteria, abundant in marine sediment and aphotic environments, have recently also been shown to produce DMSP^4,5^. These organisms produce DMSP for physiological roles in osmoprotection, sulfur and carbon storage^6^, herbivore deterrence^7^, antioxidation^8^, and hydrostatic pressure protection^9^, or as an intermediate in toxin production^10^. DMSP release into the environment through senescence, excretion, viral infection or grazing^11^ results in ubiquitous 1-100 nM levels in seawater^12^, and μM levels in marine sediment^5,9,13^, with major microbial-driven environmental impacts. Diverse microbes (photo- and heterotrophs) import DMSP for its anti-stress properties^14–17^, or as a key nutrient via catabolic pathways that provide energy and 3–10% or 50–100% of bacterial carbon^2^ or sulfur^18^ demands, respectively. DMSP catabolism is also a major biosource of climate-active gases, e.g., dimethyl sulfide (DMS), equivalent to ~300 Tg S yr^–1^ via DMSP lyases^14,19,20^, methanethiol (MeSH) through DMSP demethylation^21^, or the greenhouse gas methane^22^. DMSP and DMS are also potent foraging cues for diverse organisms^23,24^ and have key roles in global sulfur cycling and potentially climate regulation^25,26^.

**Fig. 1:**
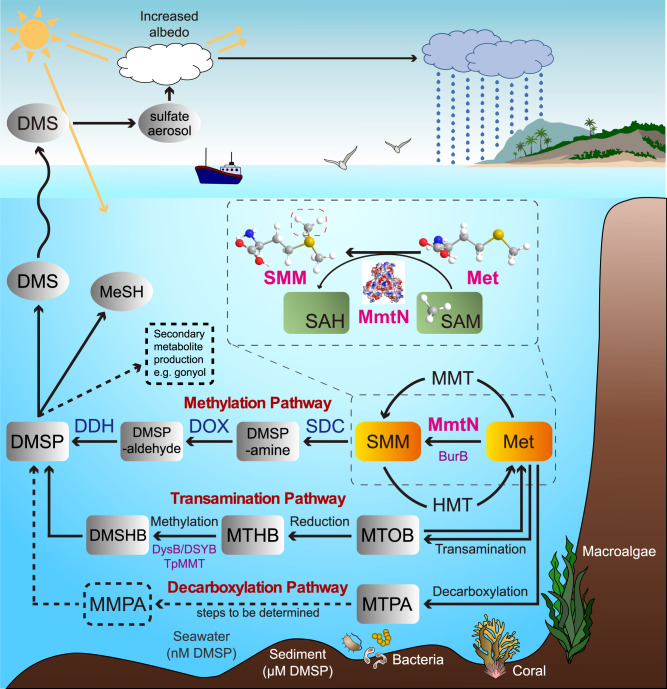
DMSP-biosynthesis pathways in the marine ecosystem. The DMSP synthesis pathways, with emphasis on the methylation pathway and in particular the *S*-methylation of Met to SMM, catalyzed by MmtN (bacteria) or MMT (plants). This step is marked by grey dotted boxes. Dotted black lines represent the unconfirmed steps of the decarboxylation pathway, and a potential but unconfirmed role for DMSP in secondary metabolite production. *S*-methyltransferase enzymes of the transamination pathway for DMSP synthesis and methylation pathway in *Burkholderia* are indicated in purple font. SMM, *S*-methylmethionine; MTOB, 4-methylthio-2-oxobutyrate; MTHB, 4-methylthio-2-hydroxybutyrate; MTPA, 3-methylthiopropylamine; DMSHB, 4-dimethylsulfonio-2-hydroxybutyrate; MMPA, methylmercaptopropionate; DMSOB, 4-dimethylsulfonio-2-oxobutyrate; MMT, Met *S*-methyltransferase; HMT, homocysteine *S*-methyltransferase; SDC, SMM decarboxylase; DOX, DMSP-amine oxidase; DDH, DMSP-aldehyde dehydrogenase.

There are three distinct pathways for DMSP synthesis from methionine (Met)^27^ (Fig. 1): the transamination pathway in marine algae, corals and many *α*-*proteobacteria*;^4,28,29^ the methylation pathway in angiosperms and some marine heterotrophic bacteria;^4,5,28,30,31^ and the decarboxylation pathway in one dinoflagellate^32^. Recently, key genes/enzymes of the transamination and methylation pathway have been identified, allowing the diversity and environmental importance of the process to be investigated via “omics” and gene probing^4,5,10,29,31,33^. In algal and bacterial DMSP synthesis pathways, the *S*-methylating enzymes DSYB/DsyB or MmtN (Fig. 1) are robust reporters of DMSP synthesis^3–5,29^.

The Met methylation pathway is initiated by two known Met *S*-methyltransferase enzymes in different organisms (Fig. 1): MmtN, the focus of this study, in several *α-* and *γ-proteobacteria* and *Actinobacteria*^5^, and BurB, only in some β-proteobacterial *Burkholderia*^10^. MmtN enzymes are missing in algae, and bacteria containing them are far less abundant in the marine water column than those microorganisms with *S*-methyltransferase enzymes of the Met transamination pathway (DsyB/DSYB, TpMMT)^4,5,29,33^. However, the abundance and transcription of *mmtN* is elevated in marine sediment environments with high DMSP concentration compared to seawater samples^5,9,13^, indicating that this methylation pathway may be important in these hotspots for DMSP production.

Despite the global abundance and importance of DMSP, there is no structural or mechanistic data on any DMSP synthesis enzymes. Here we use biochemical and structural biology methods to study MmtN driven production of *S*-methylmethionine (SMM)—the first intermediate of DMSP synthesis via the bacterial methylation pathway (Fig. 1). MmtN is an *S*-adenosyl-methionine (SAM)-dependent methyltransferase family enzyme. It is ~30% identical to the N-terminal domain of the plant Met *S*-methyltransferase MMT enzyme^34^, which is usually a larger protein (~115 kDa) because of a C-terminal aminotransferase domain, and also produces SMM, a common plant metabolite^34^.

SAM-dependent methyltransferases use SAM as the methyl donor generating *S*-adenosyl-homocysteine (SAH)^35^. Such enzymes are classified into groups based on the substrate methyl accepting atom, and comprise *O-*, *N-*, *C-*, and *S*-directed methyltransferases^35^. MmtN is an *S*-directed methyltransferase since it methylates the sulfur atom of Met.

In this work, we initially focus on the MmtN enzyme of *Roseovarius indicus* B108, a DMSP-producing roseobacter^5^ isolated from Indian Ocean deep-seawater^36^. Such roseobacters can comprise up to 20% of surface water bacteria^37^ and ~10% of bacteria in marine surface sediment^38^, and thus are abundant marine bacteria well known for their ability to cycle organosulfur compounds^14,39^. We study the enzymology of the recombinant *R. indicus* B108 MmtN and propose its catalytic mechanism based on three-dimensional structures in complex with SAM and with SAH and Met. With mechanistic insights we conduct a detailed appraisal of functional *mmtN* occurrence in diverse genomes to greatly extend the suite of organisms and environments that potentially synthesize SMM and/or DMSP. This study provides insight into the molecular mechanisms for *S*-methylation reactions in SMM and/or DMSP synthesis pathways, leading to a better understanding of an important reaction in global sulfur cycling. It implies that *mmtN*, alongside SMM and/or DMSP production, may be much more widespread than originally predicted and have previously unknown predicted functions in diverse organisms. Furthermore, given the similarity of MmtN to plant MMT sequences, this study informs on the potential mechanism of SMM formation in the plant SMM cycle.

## Results and discussion

### *R. indicus* B108 MmtN is a specific Met *S*-methyltransferase

**Fig. 2:**
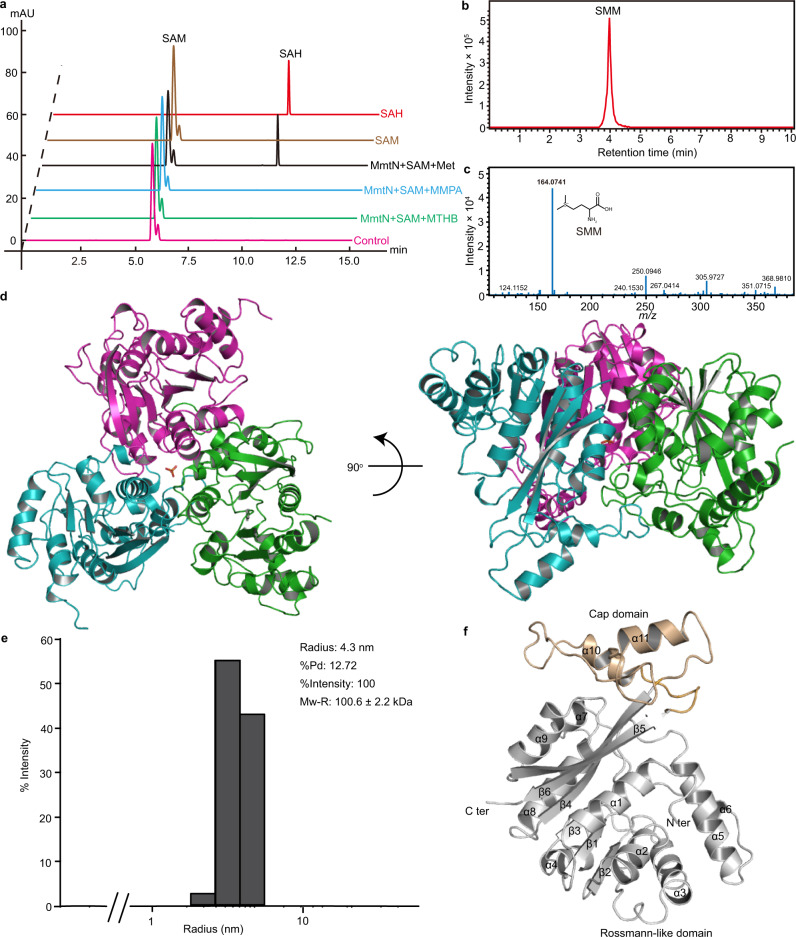
Characterization and overall structural analyses of MmtN from *R. indicus* B108. **a** The substrate specificity of *R. indicus* B108 MmtN. Detection of the methylation activities of *R. indicus* B108 MmtN towards MTHB, MMPA, and Met by HPLC at 260 nm. The reaction system without MmtN was used as the control. The data shown are representatives of triplicate experiments. **b** A total ion chromatogram (TIC) of SMM (extracted *m/z* = 164) produced by SAM-dependent MmtN *S*-methylation of Met via LC–MS. The data shown are representatives of triplicate experiments. **c** The mass spectra of SMM produced by SAM-dependent MmtN *S*-methylation of Met via LC–MS. The data shown are representatives of triplicate experiments. **d** The overall structure of MmtN. There are three MmtN molecules arranged as a trimer in an asymmetric unit. The three MmtN molecules are colored in magenta, green and cyan, respectively. The PO_4_^3-^ in MmtN is shown as orange sticks. **e** DLS analysis showing MmtN to have a molecular mass of 100.6 kDa in solution, indicating that MmtN maintains a trimer. The data shown are representatives of triplicate experiments. **f** The structure of an MmtN monomer. The cap domain is colored in orange and the rossmann-like domain in grey. Source data are provided as a Source Data file.

The *R. indicus* B108 *mmtN* gene encodes a functional 305 amino-acid Met *S*-methyltransferase^5^. The full length *mmtN* gene was synthesized and expressed in *Escherichia coli* BL21(DE3) cells. Recombinant MmtN was purified (Supplementary Fig. 1a), and its function was verified via high-performance liquid chromatography (HPLC) and liquid chromatography–mass spectrometry (LC–MS) analyses. *R*. *indicus* MmtN showed Met *S*-methylation activity, resulting in the consumption of SAM and the production of SAH (Fig. 2a) and SMM (Fig. 2b, c). The optimal pH for *R*. *indicus* MmtN enzymatic activity was ~8.0 (Supplementary Fig. 1b), and the optimal temperature was ~30 °C (Supplementary Fig. 1c), which is the same as those found for MmtN from *Novosphingobium* sp. BW1, the DMSP-producing bacterium in which *mmtN* was first identified^5^. *R*. *indicus* MmtN exhibited *K*_M_ values of 6.2 mM for SAM (Supplementary Fig. 1d), and 15.3 mM for Met (Supplementary Fig. 1e) at pH 8.0 and 30 °C. The *K*_M_ values determined in this study are higher than those reported previously for the *Novosphingobium* MmtN (2.0 mM for Met and 1.0 mM for SAM)^5^, but they are still in the same millimolar range. In accordance with findings on *Novosphingobium* MmtN, the recombinant *R. indicus* B108 MmtN only exhibited methylation activity with Met, and not MTHB or MMPA, intermediates in the transamination and decarboxylation pathways of DMSP synthesis, respectively^4,5,29^ (Figs. 1 and 2a) or glycine (Supplementary Fig. 2), a substrate for betaine synthesis. This indicates that MmtN is a specific *S*-methyltransferase for DMSP synthesis via the methylation pathway, and not the transamination or decarboxylation pathways (Fig. 1).

### MmtN consists of a cap and a Rossmann-like domain, and forms a trimer structure

X-ray crystallography was used to investigate the catalytic mechanism of MmtN Met *S*-methylation. Crystallography on wild-type *R. indicus* B108 MmtN yielded poorly diffracting crystals. Mutations of solvent-exposed charged residues by surface entropy reduction can have a positive effect on the diffraction of some protein crystals^40^, and it was in this way that MmtN variants were generated, purified and crystallized. The best diffraction data was generated using a MmtN K141A/K143A/K146A triple variant from which the crystal structure was solved to 2.5 Å resolution by the single-wavelength anomalous dispersion (SAD) method using a selenomethionine derivative (Se-derivative) (Supplementary Table 1). Importantly, this MmtN triple variant retained SAM-dependent Met *S*-methyltransferase activity, exhibiting no substantial difference to the wild-type MmtN enzyme (the specific activities of MmtN and its triple variant were 51.2 ± 1.3 U mmol^–1^ and 47.3 ± 4.5 U mmol^–1^, respectively). Thus, the triple variant and not the wild-type protein was used in all subsequent structural and mutational experiments.

MmtN crystals belong to a *P*2_1_2_1_2_1_ space group, with three molecules arranged as a trimer in the asymmetric unit (Fig. 2d). The predicted molecular mass of an MmtN monomer is 33.1 kDa (https://web.expasy.org/protparam/), and dynamic light scattering (DLS) analysis showed that the molecular mass of MmtN is 100.6 kDa (Fig. 2e), indicating that MmtN is also a trimer in solution. The MmtN trimer is assembled through interfaces between adjacent monomers, and contains an ion at its center (Fig. 2d). Ion chromatography measurements implied that the ion in MmtN was a phosphate ion (PO_4_^3-^) (Supplementary Fig. 3), consistent with the result of PO_4_^3-^ detection analysis using the PiColorLock reagent kit (Expedeon, UK)^41^. Each MmtN monomer, which is composed of six β-strands and eleven α-helices, consists of a cap domain and a Rossmann-like domain^42,43^ (Fig. 2f).

**Fig. 3:**
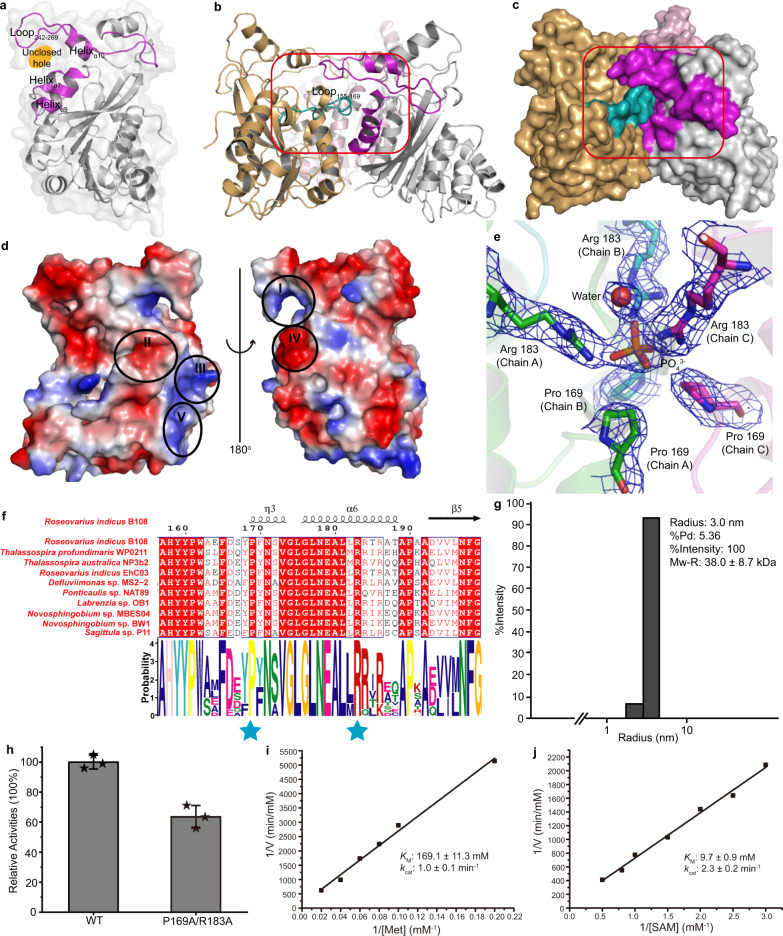
Structure and biochemical analyses of the MmtN trimer structure. **a** The structure of an MmtN monomer. Loops and helices which participate in forming the unclosed hole are highlighted in magenta. The unclosed hole is depicted in light orange. **b** Cartoon presentation of the MmtN structure. Three MmtN monomers are colored in light orange, light pink, and grey, respectively. The loop_155–169_ is highlighted in cyan. A swapped interface between neighboring monomers is marked in red box. **c** Surface presentation of the MmtN structure in **b**. **d** Surface electrostatics of an MmtN monomer. The important interfaces are marked as roman numbered circles. **e** Key residues composing the ion-binding pocket of MmtN. The PO_4_^3-^ is shown as orange sticks and water molecule as a red sphere. The 2*F*_*o*_*-F*_*c*_ densities for residues and molecules are contoured in blue-white at 1.0 σ. **f** Sequence alignment of MmtN from different marine bacteria. Residues involved in the ion-binding pocket are marked with blue stars. Sequence logos were made using The MEME Suite (https://meme-suite.org/meme/index.html). **g** DLS analysis of molecular weight of P169A/R183A variant in solution. The data shown are representatives of triplicate experiments. **h** Enzymatic activities of the P169A/R183A variant. The enzymatic activity of MmtN was taken as 100%. Data are presented as mean ± standard deviations (SD) (*n* = 3 independent experiments, indicated by stars on the bar chart). **i** Double reciprocal linear fit plots for Met methylation by the P169A/R183A variant. [Met] represents the Met concentration. The kinetic parameters of MmtN were measured at pH 8.0 and 30 °C. **j** Double reciprocal linear fit plots for SAM demethylation by the P169A/R183A variant. [SAM] represent the SAM concentration. The kinetic parameters of MmtN were measured at pH 8.0 and 30 °C. Source data are provided as a Source Data file.

In the MmtN trimer structure, loop_242–269_, helix_α7_, helix_α9_, and helix_α10_ of one monomer generate an unclosed hole (Fig. 3a), and loop_159–169_ of an adjacent MmtN monomer pops out from the Rossmann-like domain to insert into the unclosed hole (Fig. 3b), resembling a gate latch and bolt pattern (Fig. 3c). Furthermore, the interface between two adjacent monomers is composed of oppositely charged regions (Fig. 3d and Supplementary Fig. 4), suggesting a relatively stable trimer composition. To the best of our knowledge, all structures of SAM-dependent methyltransferases deposited in the Protein Data Bank are monomers, dimers and tetramers, wheras MmtN performs its catalysis as a trimer with a central ion.

To explore the catalytic mechanism of MmtN, the crystal structures of the binary complex of MmtN-SAM and the ternary complex of MmtN-SAH-Met were also solved (Supplementary Table 1 and Supplementary Fig. 5). The overall structures of MmtN-SAM and MmtN-SAH-Met were similar to that of MmtN (Supplementary Fig. 6), with a root mean square deviation (RMSD) of 0.27 Å between MmtN and MmtN-SAM complex and of 0.23 Å between MmtN and MmtN-SAH-Met complex.

### MmtN has a conserved ion-binding pocket for binding phosphate

A PO_4_^3-^ is located and likely bound, through electrostatic interactions, to a positively charged ion-binding pocket comprising residues Pro169 and Arg183 in region V of the MmtN monomers (Fig. 3d, e and Supplementary Fig. 4). Pro169 and Arg183 are strictly conserved in MmtN (Fig. 3f), but not in other deposited SAM-dependent methyltransferase structures (Supplementary Fig. 7), suggesting that this ion-binding pocket is unique to MmtN. To explore the function of the ion-binding pocket, a P169A/R183A double variant was generated. This variant appeared to have a much lower molecular weight than wild-type MmtN in gel filtration experiments (Supplementary Fig. 8a). DLS analysis also showed this, predicting a molecular mass of ~ 38.0 kDa for the P169A/R183A variant (Fig. 3g), which is consistent with it being a monomer in solution. Therefore, the ion-binding pocket is essential to stabilize the trimer MmtN structure. Moreover, these mutations decreased the enzymatic activity of MmtN (Fig. 3h), and increased the MmtN *K*_M_ values for Met (Fig. 3i) and SAM (Fig. 3j), suggesting that the trimer conformation is beneficial to anchor Met and improve the MmtN catalytic efficiency. Differential Scanning Calorimetry (DSC) measurements showed that the melting temperature (*T*_m_) of P169A/R183A variant was lower than that of WT-MmtN (Supplementary Fig. 8b), suggesting that the trimer structure also improves the thermal stability for MmtN.

### A hydrogen bond network allows MmtN to bind SAM

**Fig. 4:**
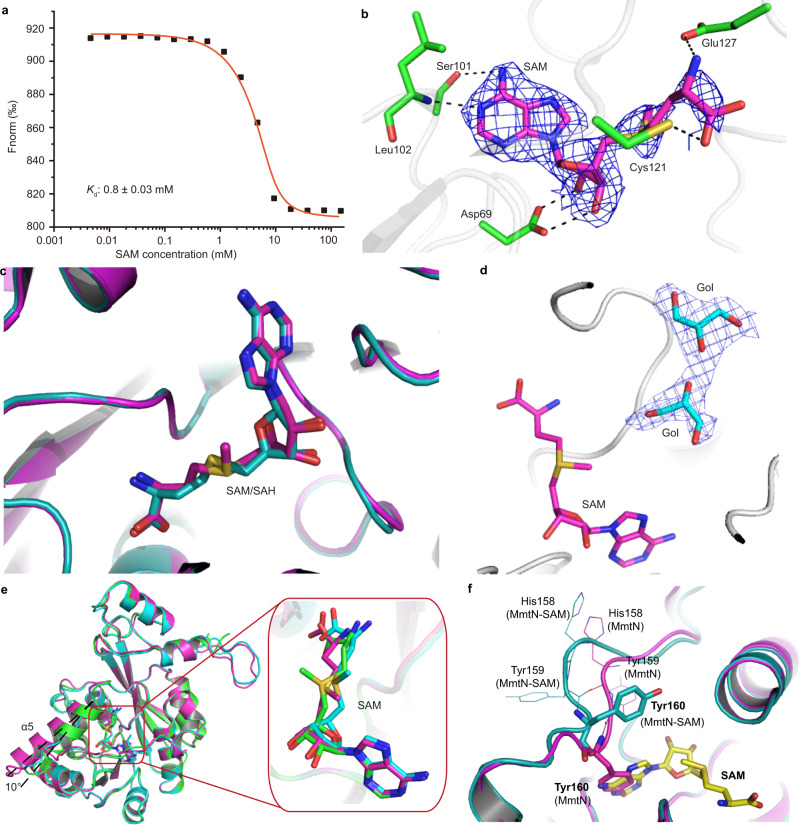
Structural analyses of important residues and molecules in the SAM binding site of MmtN. **a** MST analysis of SAM binding to MmtN. The SAM is titrated from 4.6 μM to 300 mM. The change in the thermophoretic signal leads to a *K*_d_ of 0.8 ± 0.03 mM. The data shown are representatives of triplicate experiments. **b** MmtN residues involved in SAM binding. The residues are shown as green sticks, and SAM as magenta sticks. The 2*F*_*o*_*-F*_*c*_ densities for SAM are contoured in blue at 1.0 σ. **c** Structural alignment of the MmtN-SAM complex (colored in magenta) and MmtN-SAH-Met complex (colored in cyan). The SAM molecule is shown as magenta sticks and SAH molecule as cyan sticks. **d** The locations of glycerol molecules. SAM is shown as magenta sticks, and glycerol (Gol) molecules as cyan sticks. The 2*F*_*o*_*-F*_*c*_ densities for glycerol molecules are contoured in blue-white at 1.0 σ. **e** Structural alignment of chains A, B, and C in the MmtN-SAM complex. Chains A, B, and C are colored in green, cyan and magenta, respectively. SAM molecules are shown as sticks. **f** The gating function of the flexible Loop_158–162_. MmtN-SAM binary complex is colored in cyan and MmtN in magenta. Key residues in Loop_158–162_ are labeled as cyan (MmtN-SAM complex) and magenta (MmtN) lines. Tyr 160 is shown as magenta (MmtN) or cyan (MmtN-SAM) sticks. SAM is shown as yellow sticks. Source data are provided as a Source Data file.

Microscale thermophoresis (MST) analyses indicated that SAM binds to MmtN (Fig. 4a). The electron density map of the MmtN-SAM binary complex structure showed that one SAM molecule was positioned in the active-site of each MmtN monomer, mainly through hydrogen bonding interactions (Fig. 4b). We predict Ser101 and Leu102 form hydrogen bonds with the adenine ring of SAM, whilst SAM also forms hydrogen bonds through both ribose hydroxyls to the side chain carboxyl oxygen of Asp69. The amino group of SAM contacts with the carboxyl group of Glu127 by forming another hydrogen bond. Additionally, the carboxyl group of SAM interacts with the sulfur atom of Cys121 through a hydrogen bond (Fig. 4b).

The SAH molecule in the MmtN-SAH-Met ternary complex structure was located in the same position as the SAM molecule in the binary complex structure (Fig. 4c). The electron density map of MmtN-SAM binary complex showed that two glycerol molecules were located alongside the reactive methyl group of the SAM molecule (Fig. 4d), indicating that these two glycerol molecules occupied the Met-binding site. By comparing the structures of the three monomers in the MmtN-SAM binary complex, we found that the conformations of three SAM molecules were slightly different from one another (Fig. 4e), suggesting that the binding of SAM by MmtN may not be tight. These structural observations are consistent with the biochemical data showing that MmtN had a relatively low affinity for SAM (Supplementary Fig. 1d). Moreover, the Helix_α5_ (Val131-Gln140) shifts ~10^o^ in different monomers (Fig. 4e). When superimposing the structures of MmtN and the MmtN-SAM complex, we found that Tyr160 formed a loop region (His158-Tyr162) occupying the binding site of the adenine ring of SAM (Fig. 4f), and the mutation of Tyr160 to alanine greatly increased the *K*_d_ of MmtN towards SAM (Supplementary Fig. 9). This loop swings away from the SAM-binding pocket in the MmtN-SAM complex, indicating that it has a gating function for SAM entry. Another *S*-directed methyltransferase, human thiopurine *S*-methyltransferase, also has a flexible active-site loop^44^, suggesting that the gating-functional loop of MmtN is not unique for *S*-directed methyltransferases.

### Residues Glu127, Arg132, and Glu250 are important for Met binding but are not essential for catalysis

**Fig. 5:**
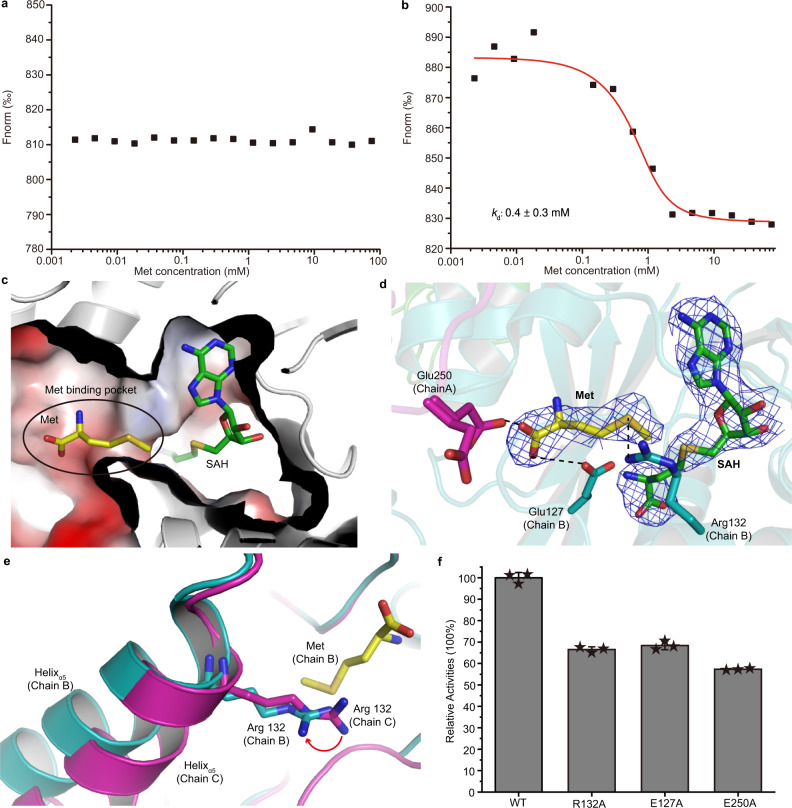
Structural analyses of important residues and molecules in the MmtN Met-binding site. **a** MST analysis of Met binding to MmtN. The Met is titrated from 2.3 μM to 75 mM. The signal/noise ratio is too low to conclude binding. The data shown are representatives of triplicate experiments. **b** MST analysis of Met binding to the MmtN-SAH complex. The Met is titrated from 2.3 μM to 75 mM. The change in the thermophoretic signal leads to a *K*_d_ of 0.4 ± 0.3 mM. The data shown are representatives of triplicate experiments. **c** Electrostatic surface of the MmtN-SAH-Met ternary complex crystal structure. SAH and Met are shown as green and yellow sticks, respectively. The Met-binding pocket is marked as a black circle. **d** Locations of SAH and Met in the active center of MmtN. The SAH and Met molecules are shown as magenta and yellow sticks, respectively. The 2*F*_*o*_*-F*_*c*_ densities for SAH and Met are contoured in blue at 1.0 σ. **e** The gating function of Helix_α5_ for Met entry. Chain B of MmtN-SAH-Met complex is colored in cyan, and chain C in magenta. Met is shown as yellow sticks. The moving direction of Arg132 is shown in red arrow. **f** Enzymatic activities of MmtN variants. The enzymatic activity of MmtN was taken as 100%. Data are presented as mean ± standard deviations (SD) (*n* = 3 independent experiments, indicated by stars on the bar chart). Source data are provided as a Source Data file.

MST analyses showed that MmtN could not bind Met (Fig. 5a), but that the MmtN-SAH complex could (Fig. 5b), suggesting that the SAM molecule must enter into the MmtN-binding site before Met is able to be bound. SAM binding likely helps the correct positioning of Met (Fig. 5c). The electron density map of MmtN-SAH-Met ternary complex showed that there was one Met molecule located near SAH in chain B (Fig. 5d), while no Met molecule was bound in chain A or chain C. The position of Met in the MmtN-SAH-Met ternary complex was similar to that of the glycerol molecules in the MmtN-SAM binary complex. In the MmtN-SAH-Met ternary complex structure, Glu127 and Glu250 (from the adjacent monomer MmtN) formed hydrogen bonds with the carboxyl group of Met. The Met sulfur atom formed a hydrogen bond with Arg132 (Fig. 5d). These hydrogen bonding interactions anchor the Met molecule near the SAH molecule in the MmtN active center. Coincidentally, the Arg132 residue comes from the relatively flexible Helix_α5_. The swing of the Helix_α5_ driving the movement of Arg132 will change the space of the Met-binding pocket (Fig. 5e), suggesting that Helix_α5_ plays a gating role for the Met entry. In addition to binding Met, structural analysis implied that Glu127 or Arg132 were possible MmtN catalytic residues, which may act as a general base to activate Met for its attack on SAM^35^. To establish the importance of these residues, E127A, R132A and E250A variants were generated, and their enzyme activities measured. All variants maintained >50% residual activity compared to MmtN lacking these substitutions (Fig. 5f), but resulted in significantly increased *K*_M_ values for Met (~8–13-fold) (Supplementary Fig. 10 and Supplementary Table 2). These data imply that Glu127, Arg132 and Glu250 are important for Met binding, and that catalytic residues may not be required for MmtN SAM-dependent Met *S*-methylation.

### A “proximity and desolvation” mechanism allows MmtN to methylate Met and generate SMM

**Fig. 6:**
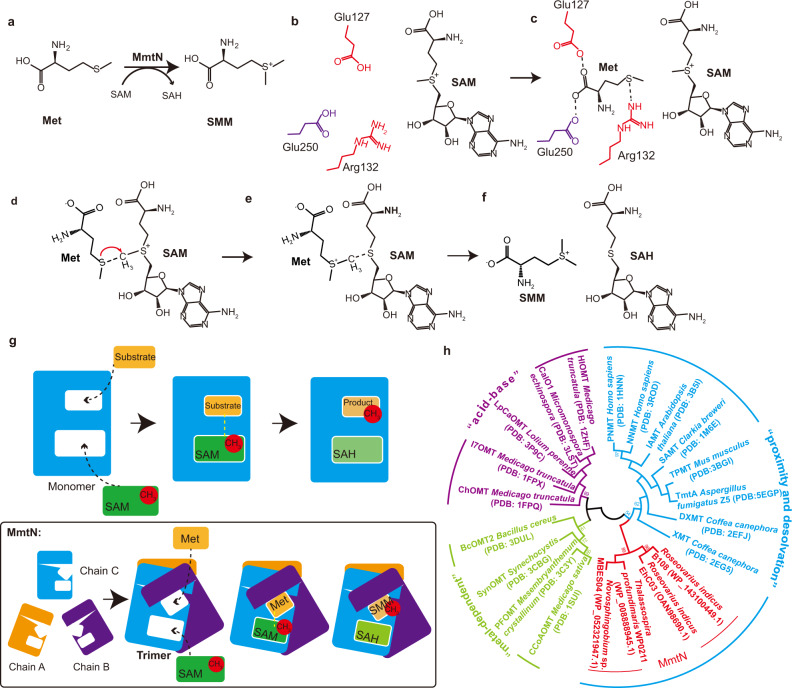
The proposed mechanism of Met methylation by MmtN. **a** The chemical reaction scheme for the MmtN-catalyzed transfer of a methyl group from SAM to Met to generate SMM and SAH. **b** The SAM molecule is firstly bound in MmtN. In the absence of Met, the Met-binding pocket is exposed. MmtN residues involved in Met binding are colored in red and purple. **c** A Met molecule enters the binding site and is positioned by Glu127, Glu250, and Arg132. **d** The Met-SAM intermediate is formed. The red arrow indicates that the sulfur atom of Met attacks the methyl group of SAM. **e** The methyl group of SAM is transferred to Met. **f** Met is methylated, and SMM and SAH are generated. **g** The schematic plot of catalysis by MmtN and other SAM-dependent methyltransferases. MmtN catalysis is shown in a black box. **h** Maximum-likelihood phylogenetic tree of MmtN homologs and other SAM-dependent methyltransferases. Phylogenetic analysis was performed using MEGA version 6.0. Source data are provided as a Source Data file.

MmtN catalyzes the *S*-methylation of Met, in which the methyl group of SAM is transferred to the Met molecule to generate SMM (Fig. 6a). There are three reported catalytic mechanisms for SAM-dependent methyltransferases: proximity and desolvation; general acid/base-mediated catalysis; and a metal-dependent mechanism^35^. Given that no general catalytic acid/base or metal ion in the active center of MmtN has been identified, it is likely that MmtN catalyzes the methyltransfer reaction using a proximity and desolvation mechanism. This method is adopted by many methyltransferases, irrespective of methyl acceptor or substrate specificity and it does not require the direct participation of enzyme catalytic groups, but rather the architecture and chemical environment of the enzyme active-site^35^. Note, the distance between the sulfur atom of Met and the active methyl group of SAM is roughly 4.5 Å in the MmtN-SAH-Met ternary complex (Fig. 5d), which is greater than the 3 Å required for methyltransfer to occur under physiological conditions^35^. Based on MST analysis, we propose that SAM entry and binding facilitates closer positioning of the Met sulfur atom towards the SAM methyl group, allowing methyltransfer (see below).

In our proposed MmtN catalytic mechanism we predict MmtN first binds SAM, and then prior to Met binding, the flexible Helix_α5_ containing Arg132 plays a gating role for Met entry (Fig. 6b). After Met enters the binding site, it is stabilized by hydrogen bonding to Glu127, Glu250 and Arg132 (Fig. 6c). In this orientation, the sulfur atom of Met is close to and attacks the active methyl group of SAM (Fig. 6d), leading to the breaking of the C–S bond of SAM and the formation of the C–S bond of Met (Fig. 6e). Finally, the generated SMM and SAH are released from the active center of MmtN (Fig. 6f).

The characterized eukaryotic SAM-dependent *S*-methyltransferases TPMT and TmtA utilize the proximity and desolvation mechanism^35,44–47^, as proposed here for MmtN. However, the TmtA and TPMT substrates are reduced gliotoxin and thiopurine prodrugs, respectively, which are very different to Met. Furthermore, the MmtN trimer structure provides a unique reaction environment that is distinct from most other known SAM-dependent methyltransferases, which, including TmtA and TPMT, are monomers (Fig. 6g). Phylogenetic analysis indicated that SAM-dependent methyltransferases are divided into three distinct clades, corresponding to the three different mechanisms (Fig. 6h). Consistent with our structural and biochemical results, MmtN homologs clustered most closely with the “proximity and desolvation” clade but they were clearly distinct from these enzymes, probably due to their unique architecture.

### The MmtN catalytic mechanism is universally conserved and likely allows SAM-dependent Met *S*-methyltransfer in other organisms

To investigate the ubiquity of the MmtN catalytic mechanism we performed a sequence alignment of MmtN proteins from different DMSP-producing bacteria (Supplementary Fig. 11). All MmtN enzymes contain: a “GxGxG” signature sequence, which is highly conserved in the SAM-dependent methyltransferase family; the SAM-binding residues (Asp69, Ser101, Leu102 and Cys121) identified here; and Arg132 for Met binding. The Glu127 and Glu250, also likely involved in Met binding, are substituted by aspartate residues in some MmtN proteins (Supplementary Fig. 11). Given glutamate and aspartate possess a similar negative charge, chemical structures and properties, the glutamate to aspartate substitution may not significantly affect Met binding. These results suggest that the proposed catalytic mechanism of MmtN has universal significance in DMSP-producing bacteria that contain MmtN.

**Fig. 7:**
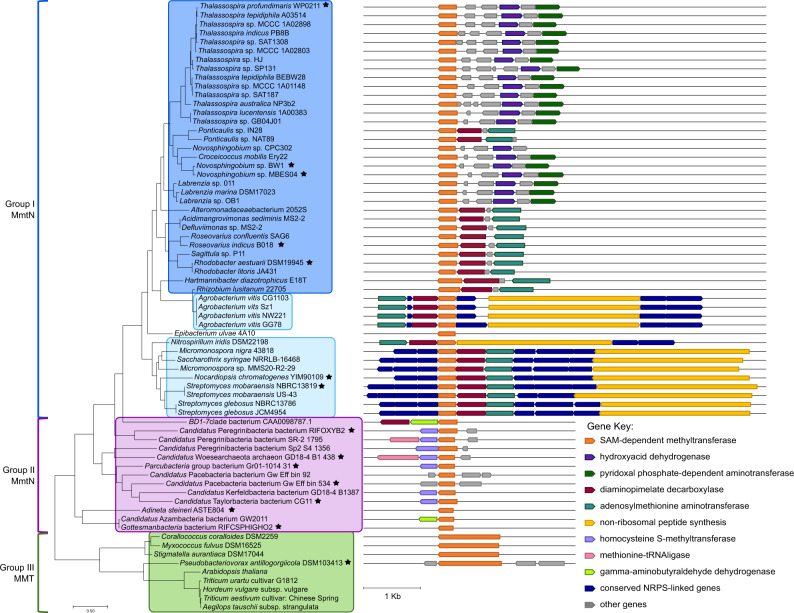
The diversity of functional MmtN family Met *S*-methyltransferases. Maximum-likelihood phylogenetic tree of MmtN family Met *S*-methyltransferases, indicating their proposed separation into three groups (I, II and III). The gene neighborhood of each *mmtN* or *MMT* gene is indicated. The two subgroups of the bacterial Group I MmtN proteins are indicated by light and dark blue boxes, defined by the presence or absence of the non-ribosomal peptide synthesis gene (yellow) linked to *mmtN*, respectively. The Group II MmtN from CPR bacteria, DPANN archaea and the animalcule *Adineta steineri* are indicated by purple boxes, and plants and bacteria containing Group III MMT are boxed in green. The phylogenetic tree is based on 65 protein sequences. Species with MmtN/MMT enzymes that have been demonstrated to be functional methionine *S*-methyltransferases are indicated by a black star. The relevant genes in the neighborhood map are indicated as arrows and are to scale. *mmtN*/*MMT* (SAM-dependent methyltransferase) genes are orange, and a color-coded key indicates the products of genes associated with it that are predicted to be involved in DMSP synthesis and/or other secondary metabolites in the respective bacteria, or in SMM cycling in Group II species. Grey arrows indicate genes that are not currently known/predicted to be involved in DMSP synthesis.

Most plants produce SMM via the plant Met *S*-methyltransferase MMT protein, but few are known to produce high levels of DMSP^34,42^. Instead, plants use MMT as a key enzyme of the SMM cycle that regulates plant SAM and Met levels^34^. MmtN is ~30% identical to the N-terminal domain of the plant MMT^34^, which is usually a larger protein (~115 kDa versus 33.06 kDa MmtN) due to an additional C-terminal aminotransferase domain^34^. Note, some *Deltaproteobacteria* and *Oligoflexia* bacteria contain a plant-like MMT, which has the same dual domain structure as plant MMT^5^ (Fig. 7). Interestingly, MMT also contains the “GxGxG” sequence, and most residues involved in binding SAM and Met are highly conserved (Supplementary Fig. 11). This implies the mechanism proposed for SAM-dependent Met *S*-methyltransfer with MmtN is also relevant for MMT enzymes, which represent an important regulatory enzyme in plants.

### MmtN is more widespread than previously considered

With a new appreciation of the MmtN structure and conserved residues we conducted a detailed survey for its presence in organisms with sequenced genomes. MmtN proteins from DMSP-producing *Actinobacteria*, *α*- and *γ*-*proteobacteria*, termed Group I, broadly cluster together and share >50% amino-acid identity (Fig. 7). To our surprise, MmtN-like proteins with ~30% identity to Group I MmtN enzymes, which we termed Group II, were detected in Metagenome Assembled Genomes (MAGs) of the archaea *Candidatus* Woesearchaeota from a groundwater metagenome (HYV86_02030 [https://www.ncbi.nlm.nih.gov/nuccore/1949109160]), numerous diverse Candidate Phyla Radiation (CPR) bacteria and the animalcule *Adineta steineri* (Fig. 7). The Group II proteins are clearly distinct from plant and bacterial MMT enzymes, termed Group III (Fig. 7).

**Fig. 8:**
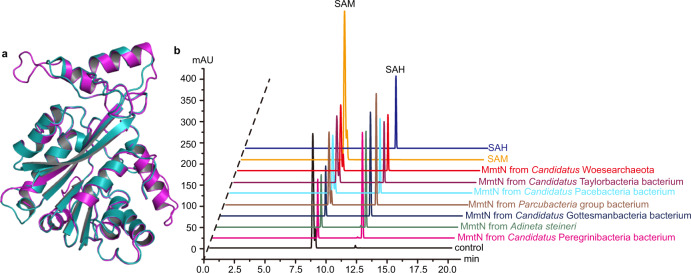
Characterization and predicted structural analyses of Group II MmtN from *Candidatus* Woesearchaeota, *Adineta steineri*, and CPR bacteria. **a** Structural alignment of MmtN from *R. indicus* B108 and *Candidatus* Woesearchaeota. The structure of MmtN from *R. indicus* B108 is colored in magenta and MmtN from *Candidatus* Woesearchaeota in cyan. The structure of MmtN from *Candidatus* Woesearchaeota was obtained using the SWISS-MODEL server (https://swissmodel.expasy.org/). **b** Detection of Met *S*-methylation activity of recombinant Group II MmtN enzymes from *Candidatus* Woesearchaeota, CPR bacteria and the animalcule *A. steineri* via the intensity of absorbance of SAM/SAH on HPLC (260 nm). The reaction system without enzymes was used as control. The data shown are representatives of triplicate experiments. Source data are provided as a Source Data file.

To infer if Group II MmtN proteins have Met *S*-methyltransferase activity the structures of representative proteins were modeled, e.g., of *Candidatus* Woesearchaeota, and found to be similar to *R. indicus* MmtN, with a RMSD of 1.31 Å (Fig. 8a), implying they catalyze the same reaction using a similar mechanism. To confirm this, the *mmtN-*like genes from *Candidatus* Woesearchaeota, *A. steineri* and 5 CPR bacteria were synthesized and demonstrated to encode functional Met *S*-methyltransferases (Figs. 7 and  8a, b). Sequence alignments revealed that these Group II MmtN enzymes contain the “GxGxG” signature sequence seen in MmtN and MMT enzymes, and most of the residues involved in binding SAM and Met are also highly conserved (Supplementary Fig. 11). Thus, it is very likely that these diverse MmtN enzymes also catalyze the methyltransfer reaction using a proximity and desolvation mechanism, with only substrate binding and no catalytic residues. With the above data, we are confident that organisms with Group II MmtN can *S*-methylate Met to SMM (Fig. 8b), a process which, until recently, was assumed to be limited to plants^5^ and Group I- and III-containing bacteria. However, we cannot confirm whether SMM is made as an intermediate in DMSP synthesis within these organisms containing Group II MmtN because in the most part they are as yet uncultivated, and we are therefore unable to determine if they produce DMSP.

### MmtN produced SMM potentially has diverse roles in Group I–III organisms

 The Group I *mmtN* gene in bacteria is either clustered with genes homologous to those from *Novosphingobium* sp. BW1 or *Streptomyces mobaraensis* (Fig. 7 and Supplementary Table 3) that likely encode downstream DMSP synthesis enzymes (Fig. 1), and all tested bacteria with these genes produced DMSP^5,31^. Bacteria with Group I MmtN can be further sub-divided according to whether their *mmtN* gene is  linked to a non-ribosomal peptide synthesis (NRPS) gene or not (Fig. 7). In the majority of these bacteria, e.g., *Novosphingobium* sp. BW1, *mmtN* is not linked to a NRPS gene.. However, for e.g., *S. mobaraensis* and *Agrobacterium vitis*, *mmtN* is likely part of a large operon with a NRPS gene similar to that associated with the *Burkholderia* Met *S*-methyltransferase *burB* gene, which is involved in producing DMSP as an intermediate in the synthesis of a cyclopropanol-based virulence factor^10^. Thus, we predict that bacteria with Group I *mmtN* and the NRPS gene may also produce DMSP as an intermediate in more complex secondary metabolite synthesis, perhaps involving gonyol^10^. Further work is required to test this hypothesis.

Archaea and CPR bacteria containing Group II *mmtN* lack strong candidate downstream DMSP synthesis genes^5,31^ and are predicted not to produce DMSP, which is largely consistent with their non-marine origin. In several of these prokaryotes, *mmtN* is closely associated with several other genes involved in Met metabolism, including those encoding a putative homocysteine *S*-methyltransferase (HMT), and, in two cases, a Met-tRNA ligase (MetRS) (Fig. 7), further supporting the role Group II MmtN likely plays in Met *S*-methylation. Furthermore, HMT and MMT (the Group III Met *S*-methyltransferase) are the key enzymes in the plant SMM cycle that controls cellular Met and SAM levels^34,48^ (Fig. 1). Thus, we predict that the diverse organisms possessing a Group II MmtN contain a SMM cycle which fulfils the same modulating role it has in plants. The Group III *MMT* in bacteria, of which only one of four strains tested (*Pseudobacteriovorax antillogorgiicola*) produced DMSP^5^, is not linked to downstream DMSP synthesis, NRPS or other Met cycling genes (Fig. 7). Thus, it is difficult to predict the potential function of SMM production in bacteria containing Group III *MMT*.

### Met *S*-methyltransferases in previously unconsidered environments

The genome of the DPANN Woesearchaeon was recovered from groundwater in California^49^, and the genomes of the CPR bacteria from various environments in the US, including a Rifle Aquifer in Colorado (*Peregrinibacteria*)^50^, Crystal Geyser in Utah (*Gottesmanbacteria* and *Taylorbacteria*)^51^ and wastewater in Germany (*Pacebacteria*). A striking common feature of these organisms with Group II *mmtN* is that they all possess small, reduced genomes and are dependent on a host for many key metabolic processes^52^. The genetic repertoire of these ultra-small episymbionts is largely shaped by their host^53^, but the hosts are entirely unknown for almost all members of the CPR/DPANN radiations. Since most CPR/DPANN microorganisms cannot produce Met, it must be imported from the host/environment. Once imported, it can be converted to SMM by MmtN, and then back to Met by HMT. An intriguing discovery is the gene encoding the putative MetRS adjacent to *mmtN* in several instances (the *Woesearchaeon* and a *Peregrinibacterium*) (Fig. 7). MetRS charges tRNA with Met, and the formylated Met-tRNA is then used in protein translation initiation in bacteria. While homologs of the CPR Met-tRNA formyltransferase are found across numerous phylogenetic phyla, homologs of the DPANN and CPR MetRS are only found in *Woesearchaeota* and *Peregrinibacteria*, respectively. This suggests that the function of MetRS in these symbiotic organisms may be different. We speculate that it could covalently link SMM to its cognate tRNA and use this AA-tRNA as an initiator tRNA, possibly as an adaptation to their symbiotic lifestyle.

To conclude, DMSP synthesis is important in stress protection, global sulfur cycling, nutrient cycles, chemotaxis, and potentially climate regulation, and the *S*-methylation step is a key reaction in the DMSP synthesis pathways^3^. The MmtN enzyme is responsible for the *S*-methylation of Met, initiating the methylation pathway for DMSP synthesis in diverse *α*- and *γ*-*proteobacteria*, and *Actinobacteria*^5,10^. In this study, the crystal structures of MmtN, MmtN-SAM binary complex, and MmtN-SAH-Met ternary complex from a deep-ocean Roseobacter were solved, and the MmtN catalytic mechanism was predicted based on structural and site-directed mutational analyses.

A mechanistic understanding of MmtN enabled the detection of functional MmtN enzymes in diverse CPR bacteria, archaea and animals never before predicted to produce SMM and/or DMSP. MmtN and plant MMT enzymes can be divided into three groups according to their sequence divergence: Group I in DMSP-producing bacteria, Group II in *Candidatus* Woesearchaeota, CPR bacteria and the animalcule *A. steineri*, and Group III (MMT) in plants and some bacteria. The Met *S*-methyltransferase catalytic mechanism predicted for Group I MmtN is likely universal in these diverse enzymes spanning all kingdoms of life.

We predict that bacteria with Group I MmtN produce DMSP either for its role in stress protection or as an intermediate in more complex secondary metabolites that involves an NRPS protein, as occurs in the production of a cyclopropanol-based virulence factor in some *Burkholderia*^10^. For this latter role, the *mmtN* and other DMSP synthesis genes are linked to a NRPS gene and are not so strictly confined to strains isolated from marine environments. It will be interesting to identify the potential metabolite/s produced from DMSP and their role in these bacteria.

We further hypothesize that diverse prokaryotes and eukaryotes containing the Group II MmtN do not produce DMSP, but rather, like plants with MMT, use this enzyme to generate SMM as part of an SMM cycle to regulate the cellular levels of Met and SAM^34,48^.

Our results provide a better understanding of how marine bacteria synthesize DMSP, particularly those bacteria in marine sediments that are hotspots for DMSP production^5,9,13^, and uncover a potentially important SMM cycle in uncultured microorganisms, animals and environments. Furthermore, our data also provides mechanistic insights into the generation of SMM from Met in plants^48^.

## Methods

### Bacterial strains and growth conditions

*E. coli* strains DH5α and BL21(DE3) were grown in lysogeny broth (LB) medium at 37 °C.

### Gene cloning, synthesis, point mutation, and protein expression and purification

The full length *mmtN* gene from *R. indicus* B108 (KRS18724.1), the *mmtN*-like gene from *Candidatus* Woesearchaeota (MBI2572614.1) and six other *mmtN* homologs (*Adineta steineri*, CAF1070771.1; *Candidatus* Peregrinibacteria bacterium, OGJ51966.1; *Candidatus* Gottesmanbacteria bacterium, OGG13690.1; *Candidatus* Pacebacteria bacterium, MBP9710772.1; *Candidatus* Taylorbacteria bacterium, PIQ68431.1; *Parcubacteria* group bacterium, TSC76298.1) were synthesized by the Beijing Genomics Institute (China), and then subcloned into the pET-22b (Novagen, America) vector with a C-terminal His tag. PCR primers were all designed with *NdeI* and *XhoI* restriction sites (Supplementary Table. 4). All of the point mutations in MmtN were introduced using the PCR-based method and were verified by DNA sequencing. Point-mutation primers for MmtN were designed using Primer 5 software (Supplementary Table 4). The MmtN proteins and all of its variants were expressed in *E. coli* BL21(DE3) cells. The cells were cultured at 37 °C in LB medium to an optical density at 600 nm (OD_600_) of 0.8–1.0 and then induced at 18 °C for 14 h with 0.5 mM isopropyl β-d-1-thiogalactopyranoside (IPTG). After induction, cells were collected by centrifugation, suspended in the lysis buffer (100 mM NaCl, 0.5% glycerol, 50 mM Tris-HCl, pH 8.0), and fractured by pressure crusher (JNBIO, China). The proteins were first purified with Ni^2+^-nitrilotriacetic acid (NTA) resin (Qiagen, Germany) and then fractionated by gel filtration on a Superdex-200 column (GE Healthcare, America). MmtN purification was carried out at 4 °C. For the Ni^2+^-NTA resin purification, wash buffer (50 mM Tris-HCl, 100 mM NaCl and 20 mM imidazole, pH 8.0) was used to remove impurified protein, then the elution buffer (50 mM Tris-HCl, 100 mM NaCl and 250 mM imidazole, pH 8.0) was used to elute the purified protein from the column. For Superdex-200 column purification, the elution buffer (10 mM Tris-HCl, 100 mM NaCl, pH 8.0) was used to elute the purified MmtN. The Se-derivative of MmtN was overexpressed in *E. coli* BL21(DE3) cells under 0.5 mM IPTG induction in M9 minimal medium supplemented with selenomethionine, lysine, valine, threonine, leucine, isoleucine, and phenylalanine. The recombinant Se-derivative was purified with the same method as that used for MmtN isolated from cells grown in rich media^54^.

### Enzymatic activity assays

The enzymatic activities of MmtN and its homologs were measured as in^5^ by detecting the production of SAH from the demethylation of SAM through its ultraviolet absorbance under 260 nm by HPLC. SAM was added in excess, and a standard curve of SAH was generated from a 1 mM stock diluted to 0–50 µM concentrations to determine SAH concentration. SAH was measured by HPLC (Ultimate 3000, Dionex and LC-20AT, Shimadzu) on a SunFire C18 column (Waters) with a linear gradient of 1–20% acetonitrile in 50 mM ammonium acetate (pH 5.5) over 24 min at 260 nm. MmtN (at a final concentration of 3.4 μM), SAM (at a final concentration of 0.6 mM) and Met, MTHB, MMPA or glycine (at a final concentration of 20 mM), were mixed with the reaction buffer containing 100 mM Tris-HCl (pH 8.0) in a total volume of 200 μl. The SAM standard was purchased from New England Biolabs (America), SAH, MTHB, and Met from Sigma-Aldrich (America), and of MMPA from Macklin Biochemical Co., Ltd (China). After the mixture was incubated at 30 °C for 30 min, the reaction was stopped by adding 10% (v/v) perchloric acid, and the amount of SAH in the reaction mixture was detected by HPLC (Ultimate 3000, Dionex, America) on a Sunfire C18 column (Waters, Ireland) with a linear gradient of 2-20% acetonitrile in 50 mM ammonium acetate (pH 5.5) over 24 min at 260 nm. To determine the optimal temperature for MmtN, the reaction mixtures were incubated at 0, 10, 20, 30, and 40 °C for 30 min. The optimal pH for MmtN was examined at 30 °C (the optimal temperature for MmtN enzymatic activity) using 200 mM Bis-Tris buffer for pH 5 to 7, 200 mM Tris buffer for pH 7 to 9, and 200 mM glycine buffer for pH 9 to 10. The control group had the same reaction system with the experimental group except that MmtN was not added. One unit of enzyme activity is defined as the amount enzyme required to produce 1 μmol SAH per minute.

### Liquid chromatography–mass spectrometry (LC–MS) analysis

LC–MS was carried out using a Dionex high-performance liquid chromatography (HPLC) system comprising a HPG-3400RS pump, a WPS-3000 autosampler, a TCC-3100 column oven, a NAD-3000RS detector and a Bruker impactHD mass spectrometer. Samples were analyzed in hydrophilic interaction chromatography (HILIC) mode using a Phenomenex Luna NH2 column (100 × 2 mm with a particle size of 3 µm) at pH 3.75. MS spray chamber conditions were as follows: capillary voltage 4.5 kV, oven temperature 30 °C, desolvation temperature 180 °C and nebulizing gas flow 1.0 L min^–1^. Solvent A was 5% acetonitrile + 95% 5 mM ammonium formate in water. Solvent B was 95% acetonitrile + 5% 100 mM ammonium formate in water. The flow rate was 0.6 ml min^–1^ and the gradient (percent solvent A/B) was *t* = 1 min, 100% B; *t* = 3.5 min, 70% B; *t* = 4.1 min, 58% B; *t* = 4.6 min, 50% B; *t* = 6.5 min, 100% B; *t* = 10 min, 100% B. The injection volume was 15 µl. The targeted mass transition corresponded to [M + H]^+^ of SMM (*m/z* = 164) in positive mode.

### Crystallization and data collection

Before crystallization, the purified MmtN K141A/K143A/K146A triple variant was concentrated to 10 mg ml^–1^ in 10 mM Tris-HCl (pH 8.0) and 100 mM NaCl. Initial crystallization trials for MmtN were performed at 18 °C using the sitting-drop vapor diffusion method. The MmtN solution was mixed with the reservoir solution with a ratio of 1:1. Diffraction-quality crystals of MmtN were obtained in hanging drops containing 0.2 M sodium acetate trihydrate (pH 7.0) and 20% (wt/vol) polyethylene glycol (PEG) 3350 at 18 °C after a 2-week incubation. Crystals of the MmtN Se-derivative were obtained in hanging drops containing 0.1 M 2-Morpholinoethanesulfonic acid monohydrate (MES) (pH 6.0), 20% (wt/vol) PEG monomethyl ether (MME) 2000 and 0.2 M NaCl at 18 °C after a 2-week incubation. To obtain the structures of the MmtN-substrates complex, MmtN was cocrystallized with SAM (5 mM) or SAH (1 mM) and Met (10 mM). Crystals of MmtN-SAM binary complex were obtained in hanging drops containing 0.2 M sodium acetate trihydrate, 0.1 M sodium cacodylate trihydrate pH 6.5 and 15% (wt/vol) PEG 8000 at 18 °C after a 2-week incubation. Crystals of MmtN-SAH-Met ternary complex were obtained in hanging drops containing 2% (vol/vol) PEG400, 0.1 M imidazole (pH 7.0) and 24% (wt/vol) PEG MME 5000 at 18 °C after a 2-week incubation. X-ray diffraction data were collected on the BL17U1, BL18U1 and BL19U1 beamlines at the Shanghai Synchrotron Radiation Facility. The initial diffraction data sets were processed using the HKL2000 and HKL3000 programs with default settings^55,56^.

### Structure determination and refinement

The crystals of MmtN, MmtN-SAM binary complex and MmtN-SAH-Met ternary complex all belonged to the *P*2_1_2_1_2_1_ space group. The structure of the MmtN Se-derivative was determined by SAD phasing. The crystal structures of MmtN, MmtN-SAM binary complex, and MmtN-SAH-Met ternary complex were determined by molecular replacement using the CCP4 program Phaser^57^ with the structure of the MmtN Se-derivative as the search model. The refinements of these structures were performed using Coot^58^ and *Phenix* ^59^. The default parameters in CCP4 Phaser, Coot, and *Phenix* were used. All the structure figures were processed using the program PyMOL (http://www.pymol.org/).

### Ion chromatography measurements

Ion chromatography measurements were performed using an Dionex ICS-6000 HPLC system (Thermo Electron, USA) operated by Chromelion software 7.2.10. To detect the negative ions in MmtN, 100 µl of MmtN protein (10 mg ml^–1^) was mixed with 300 µl 100% (v/v) acetonitrile. Sample were incubated at 30 °C for 30 min, and vaporized by rotation until acetonitrile removed completely. Samples were separated on a 4 mm Dionex^TM^ ADRS^TM^ Test Column. Conditions used were 0–3 min, 20 mM KOH (initial conditions); 3–13 min, linear gradient from 20 to 60 mM KOH; 13–18 min, 60 mM KOH; 18–18.01 min, linear gradient from 60 to 20 mM KOH; 18.01–23 min, 20 mM KOH.

### The PiColorLock assay

To detect the possible PO_4_^3-^ in MmtN, the recombinant MmtN protein was heat denatured at 100 °C and removed by centrifugation. The supematant was prepared for detection using the PiColorLock reagent kit (Expedeon, UK) according to the manufactorer protocol. Breifly, the PiColorLock reagent was supplied with a color accelerator and a special stabilizer. The “mix” was prepared shortly before the reagent was required by adding the color accelerator to the PiColorLock reagent (1% vol/vol). The mix was added to samples in a volume ratio of 1:4. After 5 min, the stabilizer (10% vol/vol) was added. The signal for the PiColorLock reagent with PO_4_^3-^ was obtained at 635 nm.

### Microscale thermophoresis (MST)-binding assay

The bindings of SAM and Met to MmtN were measured using MST method. Purified MmtN was labeled with the Large Volume Protein Labeling Kit RED-Tris-NTA 2nd Geneation (NanoTemper Technologies GmbH). Compounds were diluted in a range of concentrations and mixed with labeled MmtN at 25 °C in the buffer containing 50% vol/vol PBS (pH 7.4), 50% vol/vol TE and 0.01% Tween-20. Mixed samples were loaded into Monolith^TM^ NT.115 Series capillaries (NanoTemper Technologies GmbH), and the thermophoresis was carried out on a Monolith NT.115 instrument (NanoTemper Technologies GmbH). Binding was measured with 50% LED power and “medium” MST power, with an optimized time setting (5 s Fluo, Before; 20 s MST On; 5 s Fluo, After). *K*_d_ values were obtained by fitting the MST data in the GraphPad Prism 7 software.

### Bioinformatics

The Basic Local Alignment Search Tool (BLAST) was used to perform similarity searches through the NCBI BLAST webpage interface (https://blast.ncbi.nlm.nih.gov/Blast.cgi). CLC sequence viewer 6 and ESpript 3.0 (http://espript.ibcp.fr/ESPript/ESPript/) were used to perform multiple sequence alignments. The molecular mass of MmtN was calculated by ExPASy (https://web.expasy.org/compute_pi/). MmtN homologs and other SAM-dependent methyltransferases sequence alignmenta were carried out using BioEdit Sequence Alignment Editor with the method of ClustalW Multiple Alignment. A maximumlikelihood phylogenetic tree was constructed using MEGA version 6.0, and the tree topology was checked by 1000 bootstrap replicates. Gene maps were created using GENBANK databases visualized in Artemis^60^.

### Dynamic light scattering (DLS) analysis

DLS experiment was performed on a DynaPro NanoStar (Wyatt Technology, America) at 4 °C using 2 μl (2.5 mg ml^–1^) MmtN or P169A/R183A variant in a buffer containing 10 mM Tris-HCl, 100 mM NaCl, pH 8.0. Data were analyzed with Dynamics 71.0 software.

### Statistics and reproducibility

Data analyses were carried out using Microsoft Excel 2016, OriginPro 8.5, and OriginPro 2018C. All data shown are means ± SD. For HPLC analysis, LC–MS analysis, DLS analysis, MST analysis, SDS-PAGE analysis, ion chromatography analysis, and DSC analysis similar results were obtained from three independent experiments. Detailed data analyses are described in the text.

### Reporting summary

Further information on research design is available in the Nature Research Reporting Summary linked to this article.

## Supplementary information

Supplementary Information

Reporting Summary

## Data Availability

All data supporting the findings of this study are available within the paper (and its Supplementary Information files). All structure data generated in this study have been deposited in the Protein Data Bank (PDB) database (www.pdb.org) under accession codes 7VVV, 7VVW, and 7VVX. The sequence data of MmtN and its homologs used in this study are available in the GenBank database under accession codes KRS18724.1, MBI2572614.1, OGJ51966.1, CAF1070771.1, OGG13690.1, MBP9710772.1, PIQ68431.1, and TSC76298.1. Source data are provided with this paper.

## Source data

Source Data

## References

[1] Ksionzek KB (2016). Dissolved organic sulfur in the ocean: Biogeochemistry of a petagram inventory. Science.

[2] Simo´ R, Archer SD, Pedros-Alio C, Gilpin L, Stelfox-Widdicombe CE (2002). Coupled dynamics of dimethylsulfoniopropionate and dimethylsulfide cycling and the microbial food web in surface waters of the North Atlantic. Limnol. Oceanogr..

[3] Zhang XH (2019). Biogenic production of DMSP and its degradation to DMS-their roles in the global sulfur cycle. Sci. China Life Sci..

[4] Curson AR (2017). Dimethylsulfoniopropionate biosynthesis in marine bacteria and identification of the key gene in this process. Nat. Microbiol..

[5] Williams BT (2019). Bacteria are important dimethylsulfoniopropionate producers in coastal sediments. Nat. Microbiol.

[6] Stefels J (2000). Physiological aspects of the production and conversion of DMSP in marine algae and higher plants. J. Sea Res..

[7] Wolfe GV, Steinke M, Kirst GO (1997). Grazing-activated chemical defence in a unicellular marine alga. Nature.

[8] Sunda W, Kieber DJ, Kiene RP, Huntsman S (2002). An antioxidant function for DMSP and DMS in marine algae. Nature.

[9] Zheng Y (2020). Bacteria are important dimethylsulfoniopropionate producers in marine aphotic and high-pressure environments. Nat. Commun..

[10] Trottmann F (2020). Sulfonium acids loaded onto an unusual thiotemplate assembly line construct the cyclopropanol warhead of a burkholderia virulence factor. Angew. Chem. Int. Ed..

[11] Zubkov MV (2001). Linking the composition of bacterioplankton to rapid turnover of dissolved dimethylsulphoniopropionate in an algal bloom in the North Sea. Environ. Microbiol..

[12] Gali M, Devred E, Levasseur M, Royer SJ, Babin M (2015). A remote sensing algorithm for planktonic dimethylsulfoniopropionate (DMSP) and an analysis of global patterns. Remote Sens. Environ..

[13] Song, D. L. et al. Metagenomic insights into the cycling of dimethylsulfoniopropionate and related molecules in the eastern China marginal seas. *Front. Microbiol.* **11**, 157 (2020). 10.3389/fmicb.2020.00157 PMC7039863 32132981

[14] Curson AR, Todd JD, Sullivan MJ, Johnston AW (2011). Catabolism of dimethylsulphoniopropionate: microorganisms, enzymes and genes. Nat. Rev. Microbiol.

[15] Vila-Costa M (2006). Dimethylsulfoniopropionate uptake by marine phytoplankton. Science.

[16] Kiene RP, Linn LJ (2000). The fate of dissolved dimethylsulfoniopropionate (DMSP) in seawater: tracer studies using S-35-DMSP. Geochim. Cosmochim. Acta.

[17] Sun L, Curson ARJ, Todd JD, Johnston AWB (2012). Diversity of DMSP transport in marine bacteria, revealed by genetic analyses. Biogeochemistry.

[18] Kiene RP, Linn LJ, Bruton JA (2000). New and important roles for DMSP in marine microbial communities. J. Sea Res..

[19] Johnston AWB, Green RT, Todd JD (2016). Enzymatic breakage of dimethylsulfoniopropionate-a signature molecule for life at sea. Curr. Opin. Chem. Biol..

[20] Kettle AJ, Andreae MO (2000). Flux of dimethylsulfide from the oceans: a comparison of updated data seas and flux models. J. Geophys. Res-Atmos..

[21] Reisch CR (2011). Novel pathway for assimilation of dimethylsulphoniopropionate widespread in marine bacteria. Nature.

[22] Damm E, Kiene RP, Schwarz J, Falck E, Dieckmann G (2008). Methane cycling in Arctic shelf water and its relationship with phytoplankton biomass and DMSP. Mar. Chem..

[23] Nevitt GA (2008). Sensory ecology on the high seas: the odor world of the procellariiform seabirds. J. Exp. Biol..

[24] Seymour JR, Simo R, Ahmed T, Stocker R (2010). Chemoattraction to dimethylsulfoniopropionate throughout the marine microbial food web. Science.

[25] Mahajan AS (2015). Quantifying the impacts of an updated global dimethyl sulfide climatology on cloud microphysics and aerosol radiative forcing. J. Geophys. Res. Atmos..

[26] Charlson RJ, Lovelock JE, Andreae MO, Warren SG (1987). Oceanic phytoplankton, atmospheric sulfur, cloud albedo and climate. Nature.

[27] Dickschat JS, Rabe P, Citron CA (2015). The chemical biology of dimethylsulfoniopropionate. Org. Biomol. Chem..

[28] Gage DA (1997). A new route for synthesis of dimethylsulphoniopropionate in marine algae. Nature.

[29] Curson ARJ (2018). DSYB catalyses the key step of dimethylsulfoniopropionate biosynthesis in many phytoplankton. Nat. Microbiol..

[30] Kocsis MG, Hanson AD (2000). Biochemical evidence for two novel enzymes in the biosynthesis of 3-dimethylsulfoniopropionate in *Spartina alterniflora*. Plant Physiol..

[31] Liao C, Seebeck FP (2019). In vitro reconstitution of bacterial DMSP biosynthesis. Angew. Chem..

[32] Kitaguchi H, Uchida A, Ishida Y (1999). Purification and characterization of L-methionine decarboxylase from *Crypthecodinium cohnii*. Fish. Sci..

[33] Kageyama H, Tanaka Y, Shibata A, Waditee-Sirisattha R, Takabe T (2018). Dimethylsulfoniopropionate biosynthesis in a diatom *Thalassiosira pseudonana*: identification of a gene encoding MTHB-methyltransferase. Arch. Biochem. Biophys..

[34] Kocsis MG (2003). Insertional inactivation of the methionine S-methyltransferase gene eliminates the S-methylmethionine cycle and increases the methylation ratio. Plant Physiol..

[35] Liscombe DK, Louie GV, Noel JP (2012). Architectures, mechanisms and molecular evolution of natural product methyltransferases. Nat. Prod. Rep..

[36] Lai Q (2011). *Roseovarius indicus* sp. nov., isolated from deep-sea water of the Indian Ocean. Int. J. Syst. Evol. Microbiol..

[37] Buchan A, Gonzalez JM, Moran MA (2005). Overview of the marine Roseobacter lineage. Appl. Environ. Microbiol..

[38] Lenk S (2012). Roseobacter clade bacteria are abundant in coastal sediments and encode a novel combination of sulfur oxidation genes. Isme J..

[39] Luo HW, Moran MA (2014). Evolutionary ecology of the marine Roseobacter clade. Microbiol. Mol. Biol. R..

[40] Goldschmidt L, Cooper DR, Derewenda ZS, Eisenberg D (2007). Toward rational protein crystallization: a Web server for the design of crystallizable protein variants. Protein Sci..

[41] Wang, N. et al. Crystal structures of gamma-glutamylmethylamide synthetase provide insight into bacterial metabolism of oceanic monomethylamine. *J. Bio. Chem.* **296**, 100081 (2020). 10.1074/jbc.RA120.015952 PMC7948447 33199371

[42] Jansson A, Koskiniemi H, Mantsala P, Niemi J, Schneider G (2004). Crystal structure of a ternary complex of DnrK, a methyltransferase in daunorubicin biosynthesis, with bound products. J. Bio Chem..

[43] Grocholski T, Dinis P, Niiranen L, Niemi J, Metsa-Ketela M (2015). Divergent evolution of an atypical S-adenosyl-l-methionine-dependent monooxygenase involved in anthracycline biosynthesis. Proc. Natl Acad. Sci. USA.

[44] Peng Y (2008). Structural basis of substrate recognition in thiopurine s-methyltransferase. Biochemistry.

[45] Wu H (2007). Structural basis of allele variation of human thiopurine-S-methyltransferase. Proteins.

[46] Scheuermann TH, Lolis E, Hodsdon ME (2003). Tertiary structure of thiopurine methyltransferase from *Pseudomonas syringae*, a bacterial orthologue of a polymorphic, drug-metabolizing enzyme. J. Mol. Biol..

[47] Duell ER (2016). Sequential inactivation of gliotoxin by the *S*-methyltransferase TmtA. ACS Chem. Biol..

[48] Ranocha P (2001). The *S*-methylmethionine cycle in angiosperms: ubiquity, antiquity and activity. Plant J..

[49] He C (2021). Genome-resolved metagenomics reveals site-specific diversity of episymbiotic CPR bacteria and DPANN archaea in groundwater ecosystems. Nat. Microbiol..

[50] Anantharaman K (2016). Thousands of microbial genomes shed light on interconnected biogeochemical processes in an aquifer system. Nat. Commun..

[51] Probst AJ (2018). Differential depth distribution of microbial function and putative symbionts through sediment-hosted aquifers in the deep terrestrial subsurface. Nat. Microbiol..

[52] Castelle CJ (2018). Biosynthetic capacity, metabolic variety and unusual biology in the CPR and DPANN radiations. Nat. Rev. Microbiol..

[53] Jaffe AL (2021). Patterns of gene content and co-occurrence constrain the evolutionary path toward animal association in Candidate Phyla Radiation bacteria. mBio.

[54] Li CY (2017). Mechanistic insights into dimethylsulfoniopropionate lyase DddY, a new member of the cupin superfamily. J. Mol. Biol..

[55] Otwinowski Z, Minor W (1997). Processing of X-ray diffraction data collected in oscillation mode. Method. Enzymol..

[56] Minor W, Cymborowski M, Otwinowski Z, Chruszcz M (2006). HKL-3000: the integration of data reduction and structure solution–from diffraction images to an initial model in minutes. Acta Crystallogr. D..

[57] Winn MD (2011). Overview of the CCP4 suite and current developments. Acta Crystallogr. D.

[58] Emsley P, Lohkamp B, Scott WG, Cowtan K (2010). Features and development of Coot. Acta Crystallogr. D.

[59] Adams PD (2010). PHENIX: a comprehensive Python-based system for macromolecular structure solution. Acta Crystallogr. D..

[60] Rutherford K. (2000). Artemis: sequence visualization and annotation. Bioinformatics.

