# Supplementary material for: Insights into methionine S-methylation in diverse organisms
Source: Nat Commun. 2022 May 26;13:2947. doi: 10.1038/s41467-022-30491-5 (PMC9135737; doi:10.1038/s41467-022-30491-5)

Insights into methionine *S*-methylation in diverse organisms

Ming Peng[1,2]†, Chun-Yang Li[1]†*, Xiu-Lan Chen[2,3], Beth T. Williams[4], Kang Li[3], Ya-Nan Gao[1], Peng Wang[1], Ning Wang[2], Chao Gao[2], Shan Zhang[2], Marie C. Schoelmerich[5], Jillian F. Banfield[5], J. Benjamin Miller[4], Nick E. Le Brun[6], Jonathan D. Todd[4]*, Yu-Zhong Zhang[1,2,3]*

[1]College of Marine Life Sciences, and Frontiers Science Center for Deep Ocean Multispheres and Earth System, Ocean University of China, Qingdao, China

[2]State Key Laboratory of Microbial Technology, Marine Biotechnology Research Center, Shandong University, Qingdao, China

[3]Laboratory for Marine Biology and Biotechnology, Pilot National Laboratory for Marine Science and Technology, Qingdao, China

[4]School of Biological Sciences, University of East Anglia, Norwich Research Park, Norwich, UK.

[5]Department of Earth and Planetary Science, Innovative Genomics Institute Building, University of California, Berkeley, Berkeley, CA, USA

[6]Centre for Molecular and Structural Biochemistry, School of Chemistry, University of East Anglia, Norwich Research Park, Norwich, UK.

†Ming Peng and Chun-Yang Li contributed equally to this work.

* Corresponding author: Chun-Yang Li, Lcy@ouc.edu.cn; Jonathan D. Todd, Jonathan.Todd@uea.ac.uk; Yu-Zhong Zhang, zhangyz@sdu.edu.cn

Supplementary Information

Supplementary Figures:

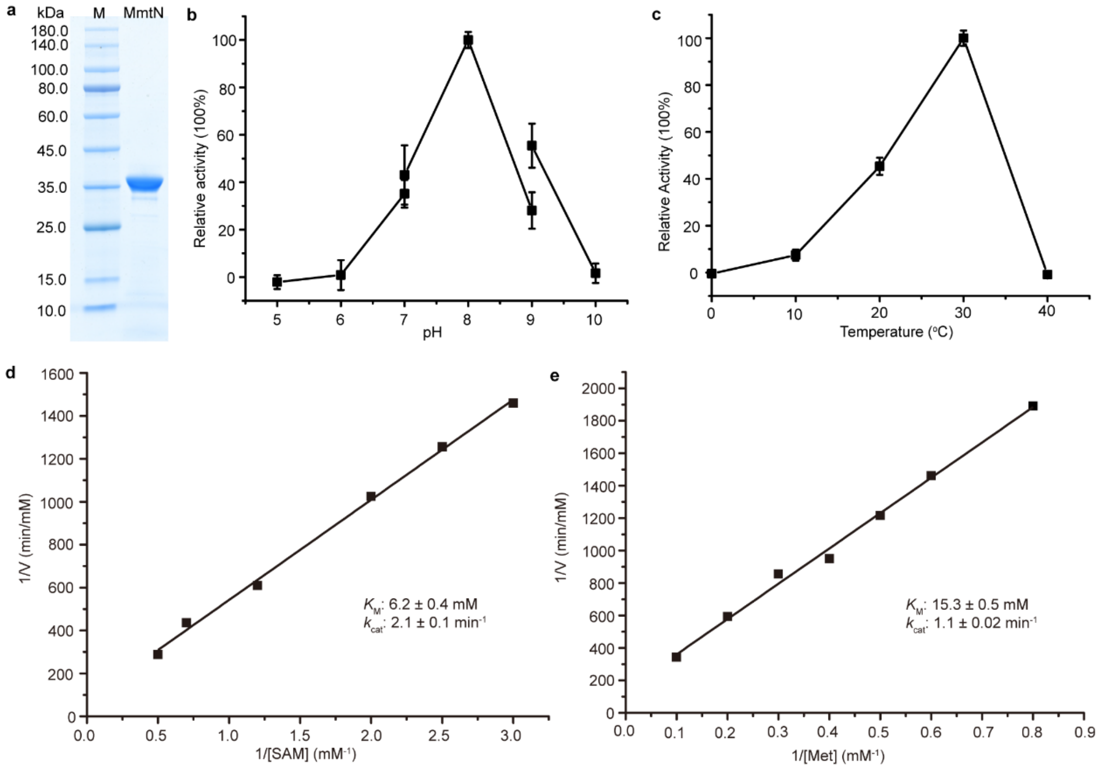

Supplementary Figure 1. Characterization of MmtN from *R. indicus* B108. (a) SDS-PAGE analysis of the purified MmtN protein. The data shown are representatives of triplicate experiments. (b) Effect of pH on the activity of MmtN. The enzymatic activity of MmtN at pH 8.0 was taken as 100%. Data are presented as mean ± standard deviations (SD) (n = 3 independent experiments). (c) Effect of temperature on the activity of MmtN. The enzymatic activity of MmtN at 30 °C was taken as 100%. Data are presented as mean ± standard deviations (SD) (n = 3 independent experiments). (d) Double reciprocal linear fit plots for SAM demethylation by MmtN. [SAM] represent SAM concentration. The kinetic parameters of MmtN were measured at pH 8.0 and 30 °C. (e) Double reciprocal linear fit plots for Met methylation by MmtN. [Met]

represent Met concentration. The kinetic parameters of MmtN were measured at pH 8.0 and 30 °C. Source data are provided as a Source Data file.

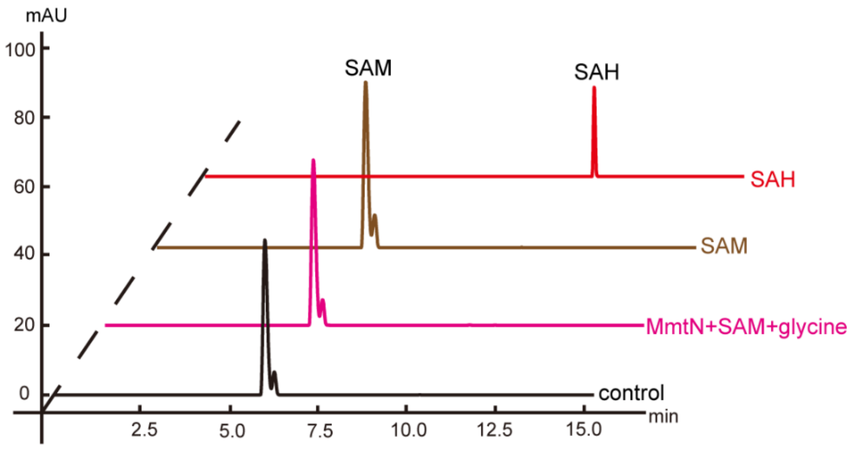

Supplementary Figure 2. Detection of the methylation activities of *R. indicus* B108 MmtN towards glycine by HPLC at 260 nm. The reaction system without MmtN was used as the control. The data shown are representatives of triplicate experiments. Source data are provided as a Source Data file.

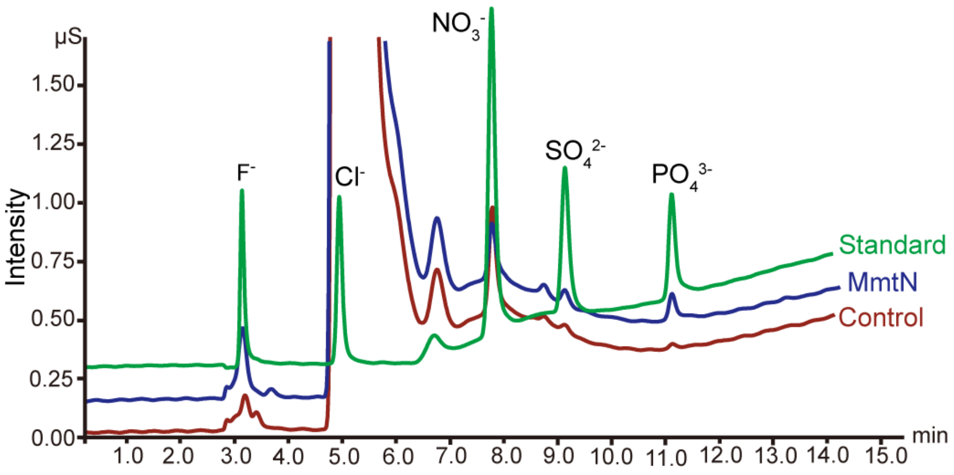

Supplementary Figure 3. Ion chromatography measurement of MmtN. The buffer without enzyme was used as the control. Note the increased PO_4^{3-} signal with the pure MmtN protein compared to the buffer control. The data shown are representatives of triplicate experiments. Source data are provided as a Source Data file.

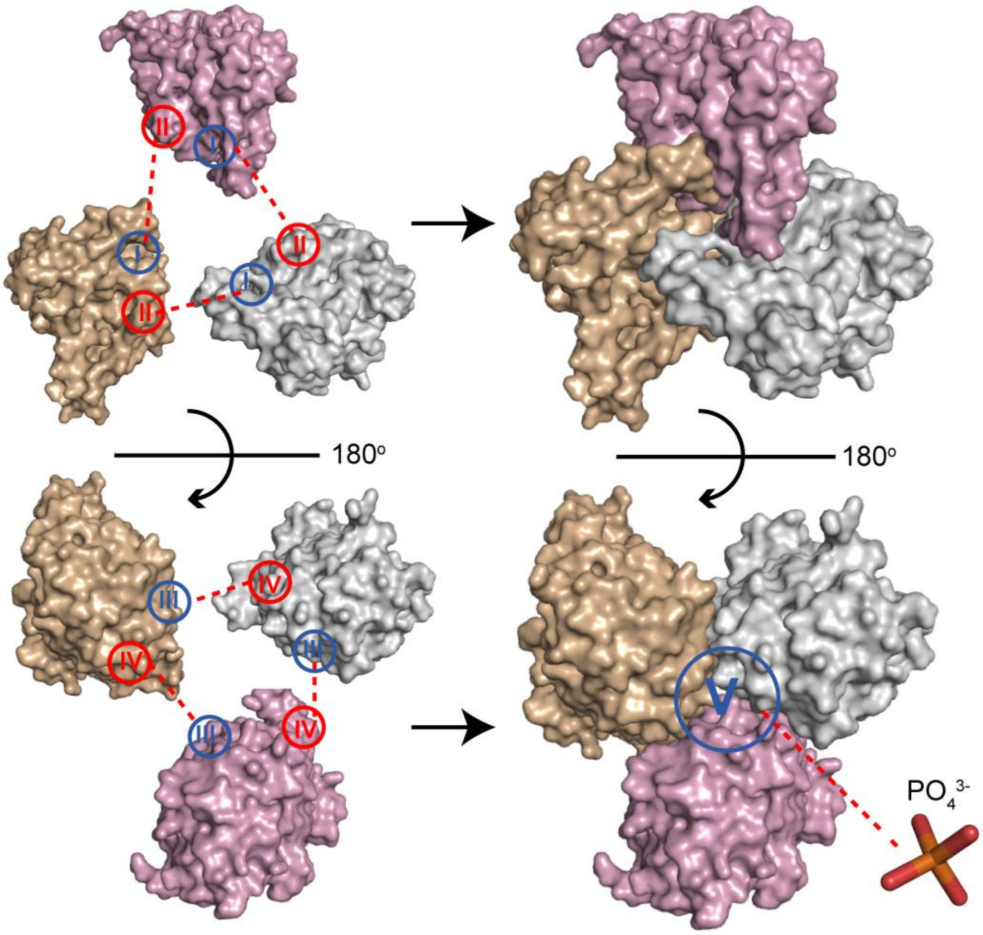

Supplementary Figure 4. The assembly of the MmtN trimer. Three MmtN monomers are colored in light orange, light pink and grey, respectively. The positively charged areas are marked as blue circles and negatively charged areas as red circles. PO_4^{3-} is shown as the orange sticks.

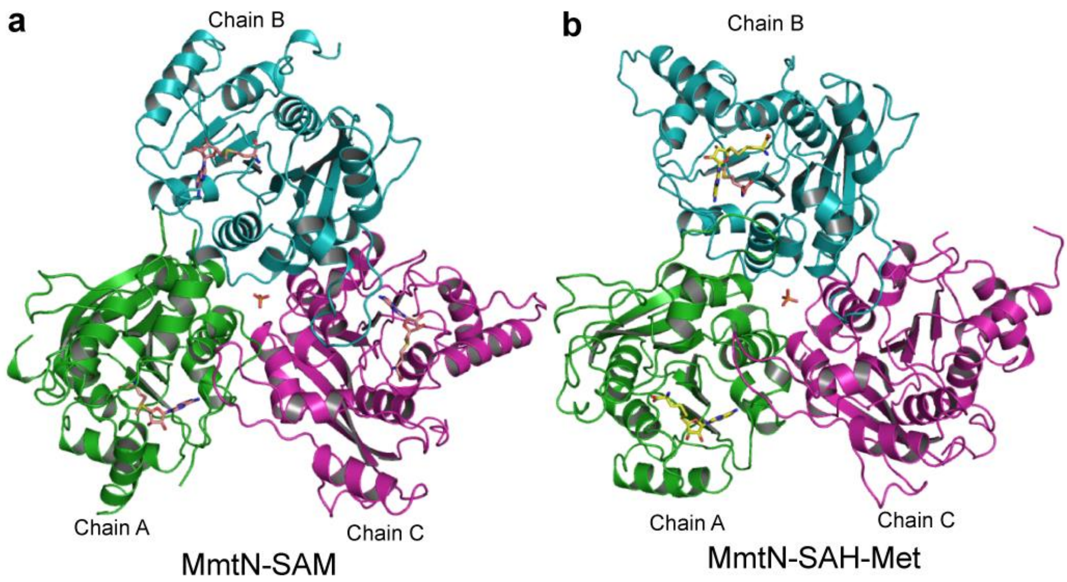

Supplementary Figure 5. Overall structures of MmtN-SAM binary complex and MmtN-SAH-Met ternary complex. (a) Overall structure of MmtN-SAM binary complex. The three MmtN molecules are colored in green, cyan and magenta respectively. The PO_4^{3-} and SAM molecules are shown as orange and salmon sticks respectively. (b) Overall structure of MmtN-SAH-Met ternary complex. The three MmtN molecules are colored in green, cyan and magenta respectively. The PO_4^{3-}, SAH and Met molecules are shown as orange, yellow and salmon sticks.

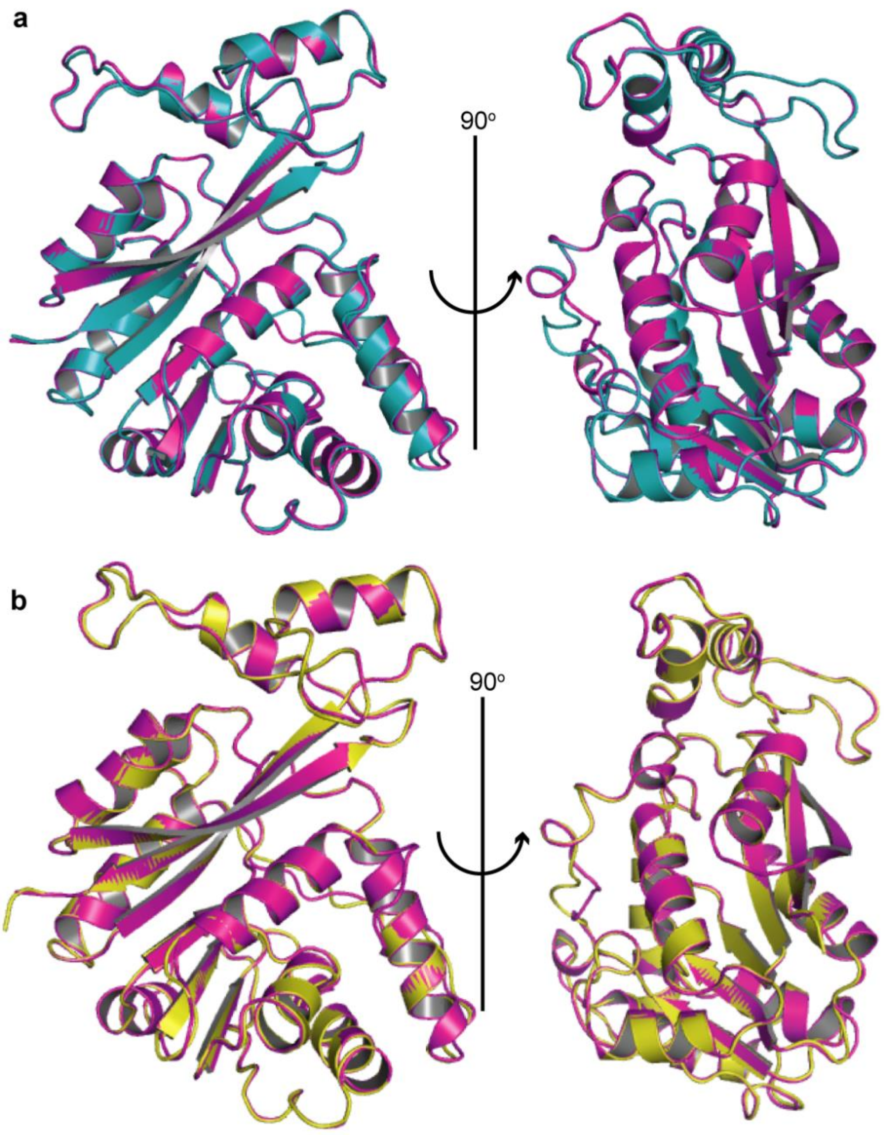

Supplementary Figure 6. Structural alignment of MmtN and MmtN-SAM/MmtN-SAH-Met complexes. (a) Structural alignment of MmtN and MmtN-SAM complex. The structure of MmtN is magenta and MmtN-SAM complex is cyan. (b) Structural alignment of MmtN and MmtN-SAH-Met complex. The structure of MmtN is magenta and MmtN-SAH-Met complex is yellow.

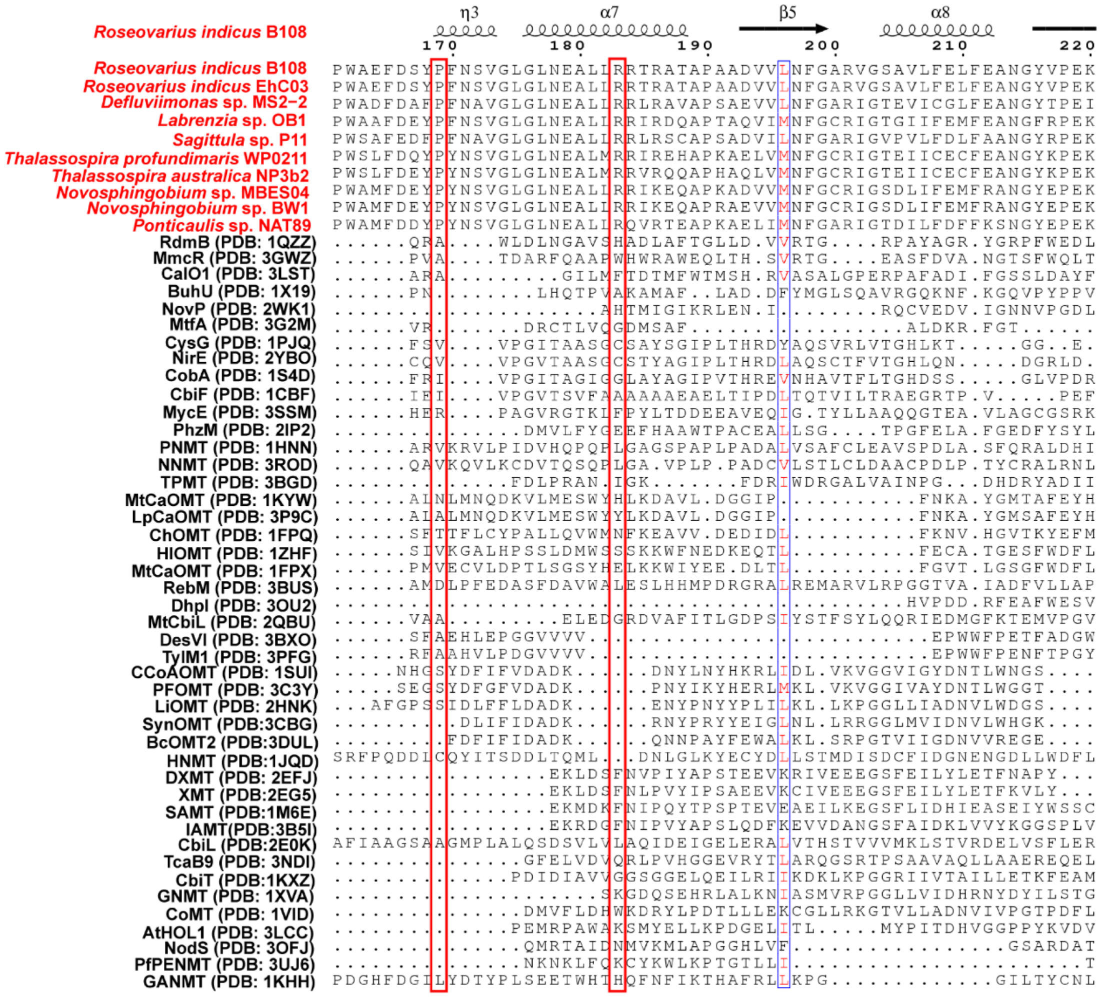

Supplementary Figure 7. Sequence analysis of residues composing the ion binding pocket in MmtN. Bacteria containing MmtN homologs are colored in red. Representative SAM-dependent methyltransferases are colored in black. Residues involved in the making of the ion binding pocket in MmtN are marked with red box. Source data are provided as a Source Data file.

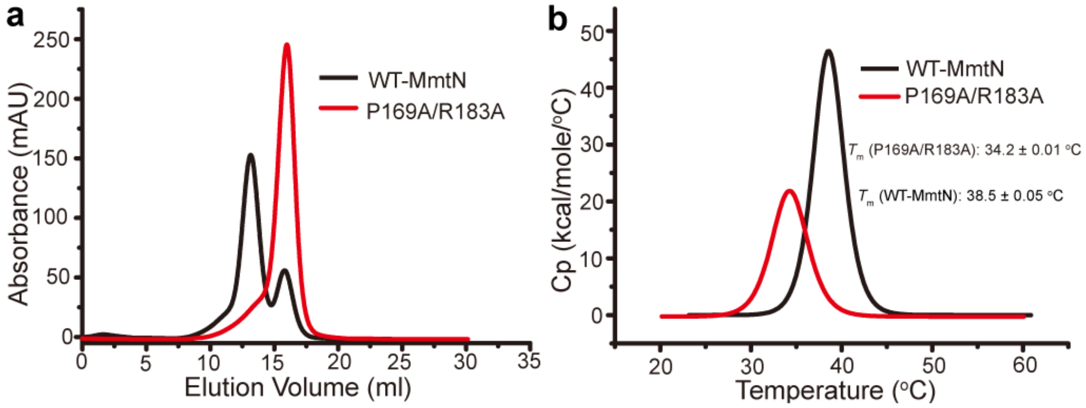

Supplementary Figure 8. Characterization of the P169A/R183A variant. (a) Gel filtration analyses of wild-type MmtN (WT-MmtN) and the P169A/R183A variant in solution. (b) The thermal stabilities of WT-MmtN and the P169A/R183A variant conformation as monitored by DSC at a scan rate of 1 °C min^{-1}. DSC traces are after subtraction of buffer scan and normalization. The data shown are representatives of triplicate experiments. Source data are provided as a Source Data file.

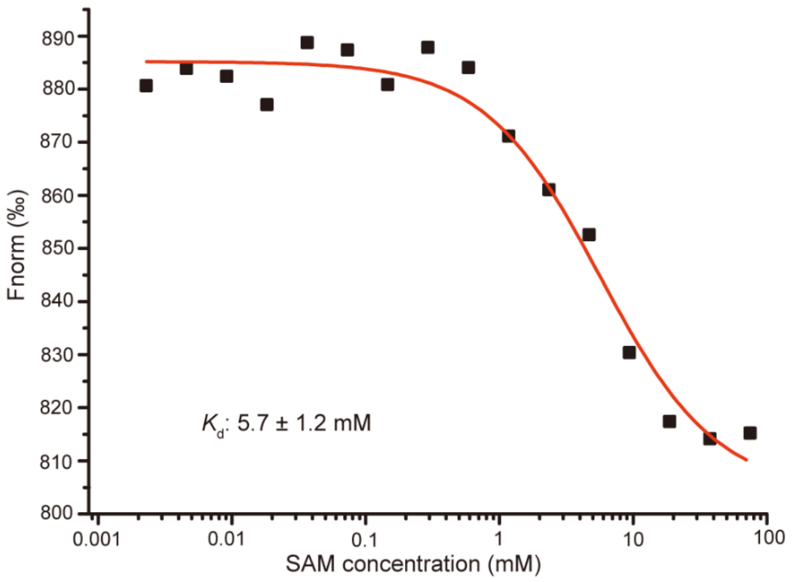

Supplementary Figure 9. The binding of the SAM to MmtN Y160A variant determined by MST. The SAM is titrated from 2.3 μM to 150 mM. The change in the thermophoretic signal leads to a K_d of 5.7 ± 1.2 mM. The data shown are representatives of triplicate experiments. Source data are provided as a Source Data file.

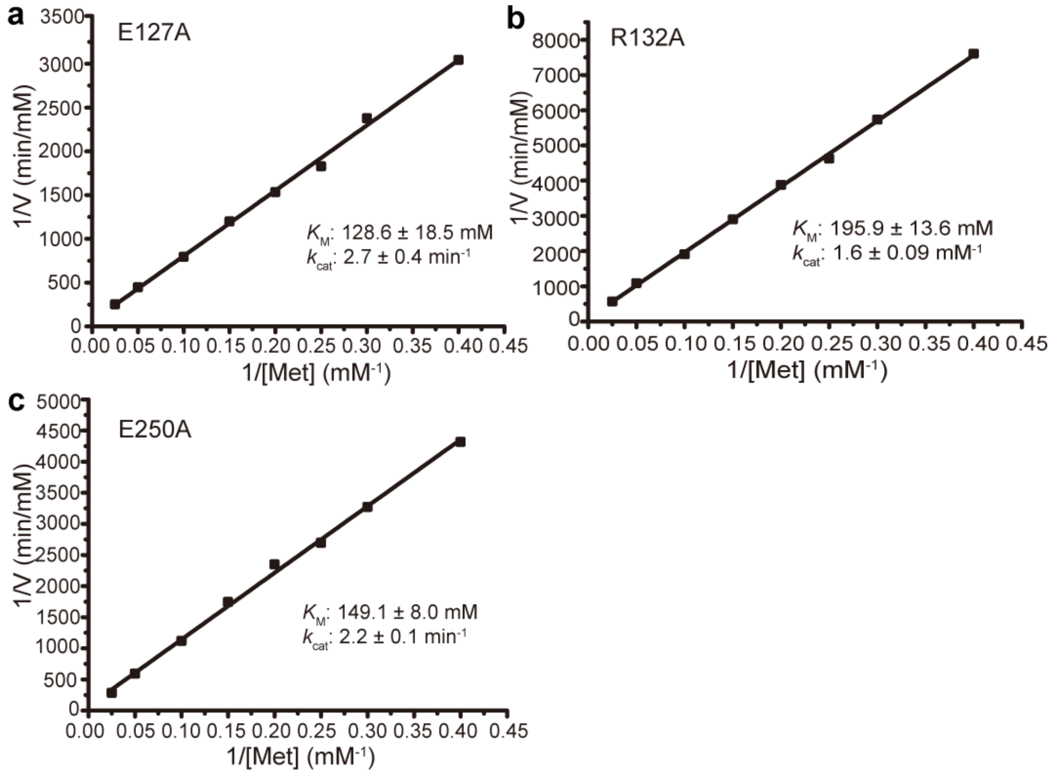

Supplementary Figure 10. Characterization of MmtN variants from *R. indicus* B108. Double reciprocal linear fit plots for Met methylation by (a) the E127A variant, (b) the R132A variant, and (c) the E250A variant. [Met] represents Met concentration. The kinetic parameters of MmtN variants were all measured at pH 8.0 and 30 °C. Source data are provided as a Source Data file.

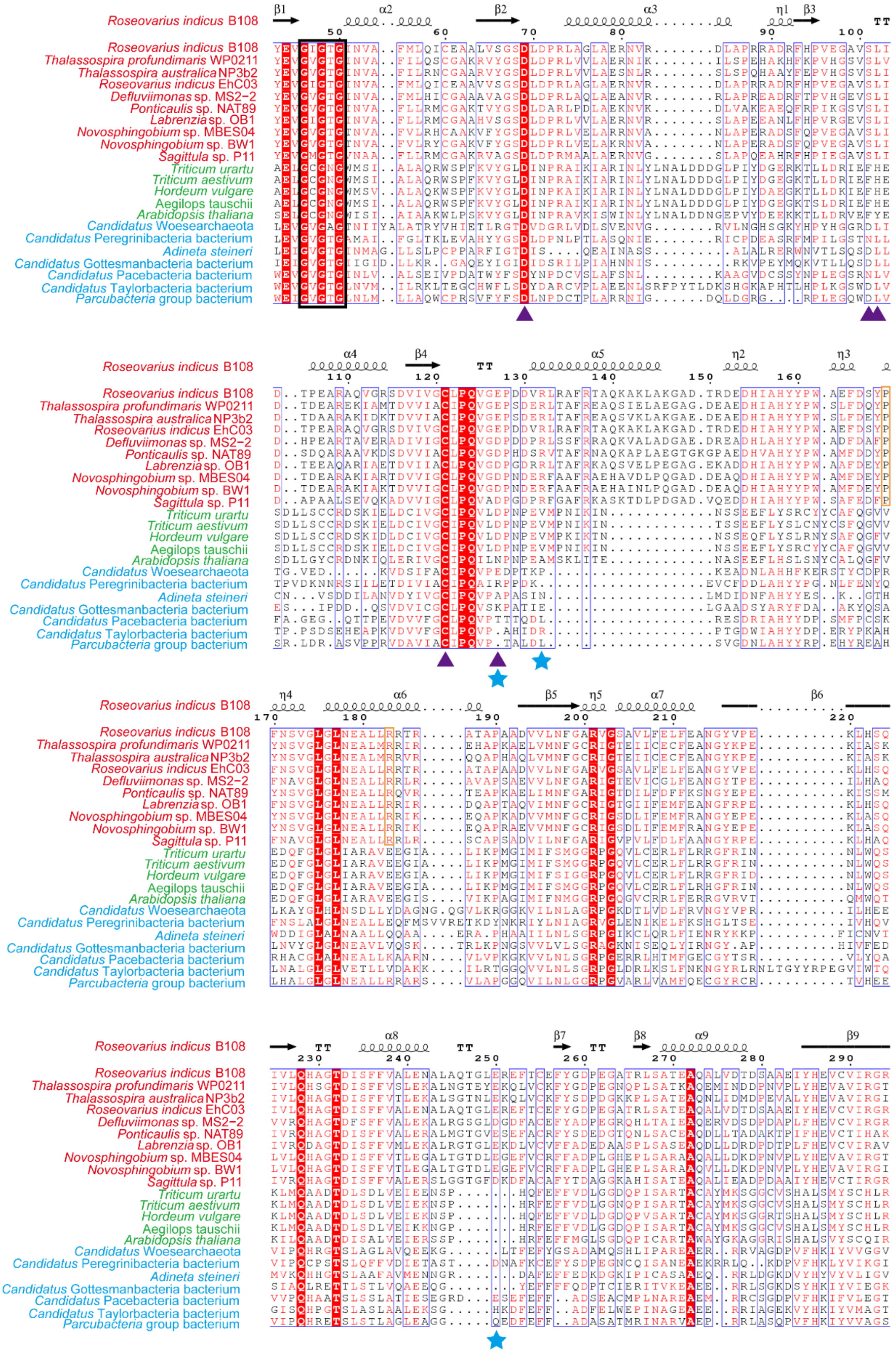

Supplementary Figure 11. Sequence alignment of group I-III MmtN polypeptides from diverse organisms. The purple triangles indicate residues which are involved in

binding SAM/SAH. Blue stars indicate Met binding residues. The “GxGxG’’ signature sequence is marked by the black box, and key residues in the ion binding pocket are marked in orange box. Bacterial group I MmtN sequences are colored in red, archaeal, bacterial or animalcule group II MmtN sequences are in blue, and the plant group III MMT sequences are in green. Source data are provided as a Source Data file.

Supplementary Tables

Supplementary Table 1. Crystallographic data collection and refinement parameters of MmtN.

Parameter	Se-derivative of MmtN	MmtN	MmtN-SAM binary complex	MmtN-SAH-Met ternary complex
**Diffraction Data**				
Space group	$P4_132$	$P2_12_12_1$	$P2_12_12_1$	$P2_12_12_1$
Unit cell				
a, b, c (Å)	169.3, 169.3, 169.3	66.7, 131.3, 134.5	62.0, 129.3 134.2	66.9, 131.1, 134.7
α, β, γ (°)	90.0, 90.0, 90.0	90.0, 90.0, 90.0	90.0, 90.0, 90.0	90.0, 90.0, 90.0
Resolution range (Å)	29.5-3.2 (3.32-3.21)[a]	47.0-2.5 (2.54-2.45)	64.65-2.11 (2.19-2.11)	41.57-2.51 (2.60-2.51)
Redundancy	71.1 (76.4)	6.3 (5.5)	12.8 (13.7)	13.0 (13.6)
Completeness (%)	99.4 (99.7)	98.6 (90.4)	98.6(99.9)	99.7 (99.8)
R_{merge}[b]	0.11 (0.54)	0.09 (0.46)	0.1 (1.2)	0.1 (1.8)
$I/\sigma I$	76.2 (15.1)	16.8 (2.2)	12.8 (2.3)	17.2 (1.9)
**Refinement statistics**				
R-factor		0.20	0.20	0.23
Free R-factor		0.23	0.23	0.25
RMSD from ideal geometry				
Bond lengths (Å)		0.02	0.02	0.02
Bond angles (°)		1.56	1.61	1.73
Ramachandran plot (%)				
Favored		94.4	95.0	89.3
Allowed		5.0	4.5	10.0
Outliers		0.6	0.5	0.7
Overall B factors (Å^2)		57.1	51.6	76.5

[a] Numbers in parentheses refer to data in the highest-resolution shell.

[b] $R_{merge} = \sum_{hkl}\sum_i |I(hkl)_i - \langle I(hkl)\rangle| / \sum_{hkl}\sum_i |I(hkl)_i$, where I is the observed intensity, $\langle I(hkl)\rangle$ represents the average intensity, and $I(hkl)_i$ represents the observed intensity of each unique reflection.

Supplementary Table 2. Kinetic parameters of MmtN variants towards Met[a].

Variants	K_M (mM)	V_{max}($\times 10^{-3}$ mM min^{-1})	k_{cat} (min^{-1})	k_{cat}/K_M (mM^{-1} min^{-1})
WT	15.3 ± 0.5	7.0 ± 0.1	1.1 ± 0.02	0.07
E127A	128.6 ± 18.5	17.2 ± 2.3	2.7 ± 0.4	0.02
R132A	195.9 ± 13.6	10.5 ±0.6	1.6 ± 0.1	0.01
E250A	149.1 ± 8.0	13.9 ± 0.6	2.2 ± 0.1	0.01

[a] The data shown in the table are from triplicate experiments (means ± standard deviations).

Supplementary Table 3. The different suites of genes associated with *mmtN* in *Novosphingobium* sp. BW1 and *Streptomyces mobaraensis* that likely encode for downstream enzymes in the DMSP methylation production pathway.

Organism	Gene Name	Accession Number
Streptomyces mobaraensis	class I SAM-dependent methyltransferase (*mmtN*)	WP_004946341.1
	PLP-dependent diaminopimelate decarboxylase	WP_004946345.1
	DMSP aminotransferase	WP_040890262.1
	DMSP aldehyde dehydrogenase	WP_004945985.1
Novosphingobium sp. BW1	class I SAM-dependent methyltransferase (*mmtN*)	WP_148626858.1
	hydroxyacid dehydrogenase	WP_148626855.1
	pyridoxal phosphate-dependent aminotransferase	WP_148626853.1

Supplementary Table 4. Oligonucleotide primers used in this study.

Primer name	sequence (5’ to 3’)	Purpose
mmtN_NdeI-F	AAGAAGGAGATATACATATGATGGGTGACAGCGAAGAACCG	Cloning of *R. indicus* B108 *mmtN* into pET-22b.
mmtN_XhoI-R	TGGTGGTGGTGGTGCTCGAGGCTATCACTCGGATCATTTT	Cloning of *R. indicus* B108 *mmtN* into pET-22b.
MmtN(K141A)-F	CTTTTGCCAGTTTGGCTGCCTGTGCGGTGCGAAAGG	Point-mutation of MmtN Lys141 to Ala.
MmtN(K141A)-R	CCTTTCGCACCGCACAGGCAGCCAAACTGGCAAAAG	Point-mutation of MmtN Lys141 to Ala.
MmtN K141A(K143A)-F	GCACCTTTTGCCAGTGCGGCTGCCTGTGCGGT	Point-mutation of MmtN K141A variant Lys143 to Ala.
MmtN K141A (K143A)-R	ACCGCACAGGCAGCCGCACTGGCAAAAGGTGC	Point-mutation of MmtN K141A variant Lys143 to Ala.
MmtN K141A K143A(K146A)-F	TATCTGCACCTGCTGCCAGTGCGGCTGCCTGTGC	Point-mutation of MmtN K141A/K143A variant Lys146 to Ala.
MmtN K141A K143A(K146A)-R	GCACAGGCAGCCGCACTGGCAGCAGGTGCAGATA	Point-mutation of MmtN K141A/K143A variant Lys146 to Ala.
MmtN(E127A)-F	GCACATCATCCGGTGCACCAACCTGCGGC	Point-mutation of MmtN Glu127 to Ala.
MmtN(E127A)-R	GCCGCAGGTTGGTGCACCGGATGATGTGC	Point-mutation of MmtN Glu127 to Ala.
MmtN(R132A)-F	CGAAAGGCACGCAGGGCCACATCATCCGGTTC	Point-mutation of MmtN Arg132 to Ala.
MmtN(R132A)-R	GAACCGGATGATGTGGCCCTGCGTGCCTTTCG	Point-mutation of MmtN Arg132 to Ala.
MmtN(E250A)-F	CAAGTGAATTCACGTGCCAGGCCGGTCTGTG	Point-mutation of MmtN Glu250 to Ala.
MmtN(E250A)-R	CACAGACCGGCCTGGCACGTGAATTCACTTG	Point-mutation of MmtN Glu250 to Ala.
MmtN(P169A)-F	CAACGCTATTAAACGCATAGCTATCAAATTCTGCCCACG	Point-mutation of MmtN Pro169 to Ala.
MmtN(P169A)-R	CGTGGGCAGAATTTGATAGCTATGCGTTTAATAGCGTTG	Point-mutation of MmtN Pro169 to Ala.
MmtN P169A(R183A)-F	GCGCGGGTACGGGCCAGCAGGGCTTC	Point-mutation of MmtN Pro169 variant Arg183 to Ala.
MmtN P169A(R183A)-R	GAAGCCCTGCTGGCCCGTACCCGCGC	Point-mutation of MmtN Pro169 variant Arg183 to Ala.

MmtN(Y160A)-F	ATTCTGCCCACGGAGCATAATGGGCAATATGATCTTCATCACG	Point-mutation of MmtN Tyr160 to Ala.
MmtN(Y160A)-R	CGTGATGAAGATCATATTGCCCATTATGCTCCGTGGGCAGAAT	Point-mutation of MmtN Tyr160 to Ala.

Uncropped scans of SDS-PAGE in Supplementary Figure 1a.

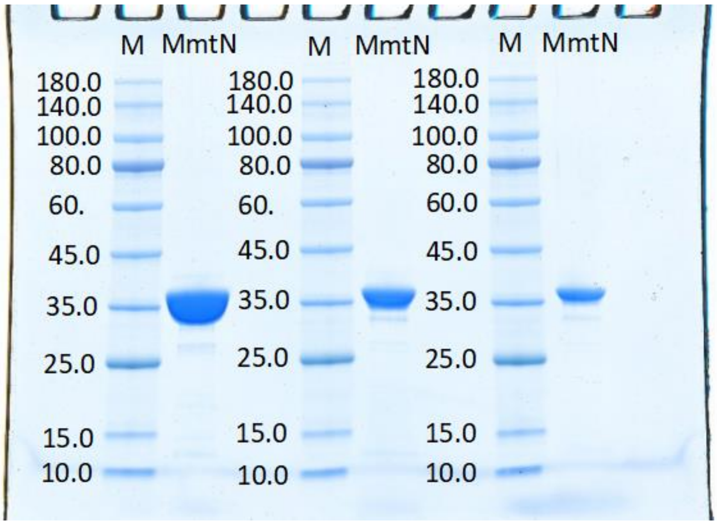

Supplement: Supplementary file 1 — Supplementary Information [file 41467_2022_30491_MOESM1_ESM.pdf]
